# Genetic architecture controlling variation in grain carotenoid composition and concentrations in two maize populations

**DOI:** 10.1007/s00122-013-2179-5

**Published:** 2013-09-17

**Authors:** Catherine B. Kandianis, Robyn Stevens, Weiping Liu, Natalia Palacios, Kevin Montgomery, Kevin Pixley, Wendy S. White, Torbert Rocheford

**Affiliations:** 1 Department of Crop Sciences, University of Illinois, Urbana, IL 61801 USA; 2 Department of Pediatrics, USDA-ARS Children’s Nutrition Research Center, Baylor College of Medicine, Houston, TX 77030 USA; 3 U.S. Agency for International Development, Washington, DC 20523 USA; 4 Department of Food Science and Human Nutrition, Iowa State University, Ames, IA 50011 USA; 5 International Maize and Wheat Improvement Center (CIMMYT), Apdo Postal 6-641, 06600 Mexico, DF Mexico; 6 Montgomery Consulting, Maroa, IL 61756 USA; 7 Department of Agronomy, Purdue University, West Lafayette, IN 47907 USA

## Abstract

*****Key message***:**

**Genetic control of maize grain carotenoid profiles is coordinated through several loci distributed throughout three secondary metabolic pathways, most of which exhibit additive, and more importantly, pleiotropic effects.**

**Abstract:**

The genetic basis for the variation in maize grain carotenoid concentrations was investigated in two F_2:3_ populations, DEexp × CI7 and A619 × SC55, derived from high total carotenoid and high β-carotene inbred lines. A comparison of grain carotenoid concentrations from population DEexp × CI7 grown in different environments revealed significantly higher concentrations and greater trait variation in samples harvested from a subtropical environment relative to those from a temperate environment. Genotype by environment interactions was significant for most carotenoid traits. Using phenotypic data in additive, environment-specific genetic models, quantitative trait loci (QTL) were identified for absolute and derived carotenoid traits in each population, including those specific to the isomerization of β-carotene. A multivariate approach for these correlated traits was taken, using carotenoid trait principal components (PCs) that jointly accounted for 97 % or more of trait variation. Component loadings for carotenoid PCs were interpreted in the context of known substrate-product relationships within the carotenoid pathway. Importantly, QTL for univariate and multivariate traits were found to cluster in close proximity to map locations of loci involved in methyl-erythritol, isoprenoid and carotenoid metabolism. Several of these genes, including *lycopene epsilon cyclase*, *carotenoid cleavage dioxygenase1* and *beta*-*carotene hydroxylase*, were mapped in the segregating populations. These loci exhibited pleiotropic effects on α-branch carotenoids, total carotenoid profile and β-branch carotenoids, respectively. Our results confirm that several QTL are involved in the modification of carotenoid profiles, and suggest genetic targets that could be used for the improvement of total carotenoid and β-carotene in future breeding populations.

**Electronic supplementary material:**

The online version of this article (doi:10.1007/s00122-013-2179-5) contains supplementary material, which is available to authorized users.

## Introduction

**Fig. 1:**
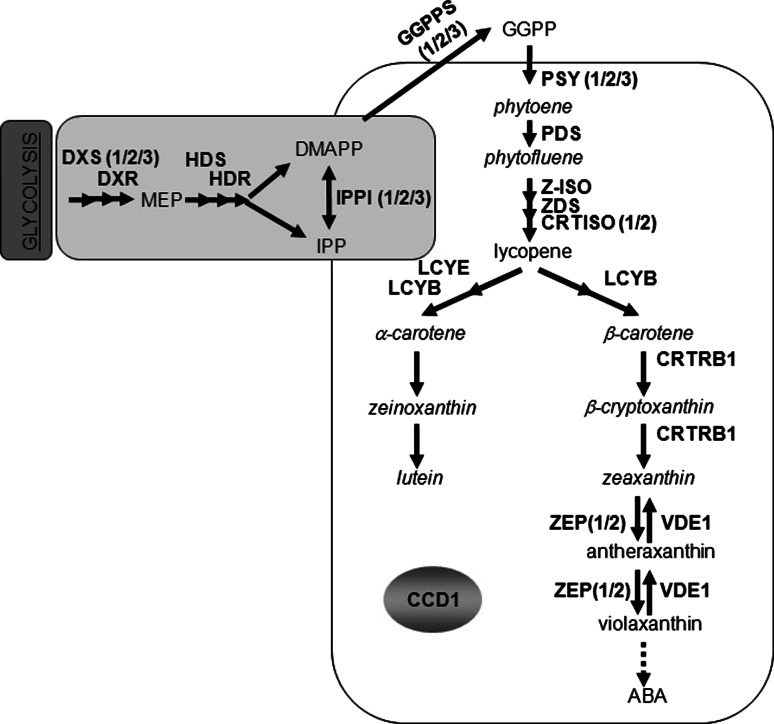
Biochemical pathways contributing to carotenoid biosynthesis. Carotenoids are derived from products of glycolysis (*dark gray fill*) and isoprenoid biosynthesis (*light gray fill*). Substrate is committed to the carotenoid biosynthesis pathway (*white fill*) by phytoene synthase (PSY). Pathway enzymes are shown in *bold*, carotenoid intermediates measured in this study in *italics*, and all other intermediates in *normal font*. Enzymes defined as: *DXS* 1-deoxy-d-xylulose-5-phosphate synthase, *DXR* DXP reductoisomerase, *HDS* 4-hydroxy-3-methylbut-2-en-1-yl diphosphate synthase, *HDR* 4-hydroxy-3-methylbut-2-en-1-yl diphosphate reductase, *IPPI* isopentyl pyrophosphate isomerase, *GGPPS* geranyl geranyl pyrophosphate synthase, *PSY* phytoene synthase, *PDS* phytoene desaturase, *Z-ISO* 15-*cis* zeta carotene isomerase, *ZDS* zeta carotene desaturase, *CRTISO* carotenoid isomerase, *LCYε* lycopene epsilon cyclase, *LCYβ* lycopene beta cyclase, *CRTRB1* beta-carotene hydroxylase, *ZEP* zeaxanthin epoxidase, *VDE1* violaxanthin de-epoxidase, *CCD1* carotenoid cleavage dioxygenase 1. Abbreviated intermediates are: *MEP* methyl-erythritol 4-phosphate, *DMAPP* dimethylallyl diphosphate, *IPP* isopentyl diphosphate, *GGPP* geranyl geranyl pyrophosphate. References and genetic location for all pathway genes are provided in Supplemental Table 7

Selection of yellow grain varieties is noted as a hallmark of maize domestication (Doebley et al. 2006). Yellow pigmentation is attributed to an accumulation of carotenoids in the endosperm resulting from a gain of function mutation in the first, rate-limiting enzyme in the carotenoid pathway, phytoene synthase (*y1*, *psy1*) (Palaisa et al. 2004) (Fig. 1). In the 1930s, the discovery of increased nutrition in yellow maize grain (Mangelsdorf and Fraps 1931) led to selection of pigmented grain as a desirable quality trait for both human food and animal feed (Bauernfeind et al. 1981; Weber 1987). It is now widely known that plant-based carotenoids, the source of this yellow pigment in maize grain, can provide dietary vitamin precursors and antioxidants that are essential to human health.

Provitamin A (proVA) carotenoids form the retinyl structural base for vitamin A molecules through cleavage reactions specific to animal metabolism (Fierce et al. 2008; Leuenberger et al. 2001). ProVA carotenoids can be found in many plant-based foods and include β-carotene (2 retinyl groups), β-cryptoxanthin (1 retinyl group) and α-carotene (1 retinyl group). Lutein and zeaxanthin, dihydroxy-xanthophyll conversion products of these proVA carotenoids in plants, are also prevalent in vegetative and seed tissues and are an abundant source of antioxidants. ProVA carotenoids provide vitamin A activity which is involved in immune function, protection of vision systems, and cellular differentiation (Stephensen 2001; Whitcher et al. 2001), and dihydroxy-xanthophylls are believed to aid in the prevention of macular degeneration and visual acuity (Chucair et al. 2007; Seddon et al. 1994), prompting the implementation of various nutritional interventions involving increased consumption of plant-based carotenoids. Consumption of several of these plant metabolites is associated with research outcomes indicative of health improvement (Bouis et al. 2011; Li et al. 2010; Tang et al. 2009). Therefore, from a health and nutritional standpoint both the quantity and quality of carotenoid profiles are important when plant-based foods are being used to satisfy dietary requirements.

Maize grain carotenoid concentrations are among the highest produced in cereals (Howitt and Pogson 2006), and exhibit considerable diversity in the composition of grain carotenoid profiles with respect to the predominant carotenoids (lutein and zeaxanthin), proVA carotenoids (α-carotene, β-carotene and β-cryptoxanthin) and other non-proVA carotenoids (zeinoxanthin) (Harjes et al. 2008). Genetic analyses conducted with populations segregating in seed color (Chandler et al. 2013; Islam 2004) and carotenoid profiles (Chander et al. 2008; Stevens 2007; Wong et al. 2004) have demonstrated that compositional and concentration differences in seed carotenoids are quantitatively inherited. Biochemical characterization of various maize endosperm color mutants originally classified by Robertson (1975) has helped to identify various maize-specific homologs of carotenoid pathway genes reported in bacteria or model crop species (Fig. 1). Interestingly, concomitant decreases in lutein and zeaxanthin are observed when selection for increased ProVA is imposed with crtRB1 (Yan et al. 2010; data not shown), indicating that the accumulation of carotenoid metabolites can be competitive due to a related biochemical origin or to an altered flux through the pathway.

Genetic mapping studies have also indicated that a significant number of the QTL involved with carotenoid accumulation in grain exhibit pleiotropic effects on multiple carotenoid traits (Harjes et al. 2008; Yan et al. 2010). This suggests that these QTL could control the substrate-product conversion of multiple metabolites in the pathway, therein revealing potential pathway control points. Reactions involved in glycolysis and isoprenoid biosynthesis appear to have a large impact on the magnitude of carotenoid accumulation in a manner similar to PSY1 (Estevez et al. 2001; Lois et al. 2000; Sandmann et al. 2006). Carotenoid production occurs within plastid organelles, in close proximity to the site of glycolysis. As depicted in Fig. 1, three-carbon molecules from glycolysis are committed to the methyl-erythritol phosphate (MEP) pathway through deoxyxylulose synthase (DXS) and deoxyxylulose reductase (DXR) (Lichtenthaler et al. 1997). The MEP pathway synthesizes five-carbon isoprenoids and subsequently produces isoprene isomers to form a common substrate pool shared by secondary metabolite pathways synthesizing plant sterols, terpenes, gibberellins, and carotenoids (Estevez et al. 2001).

Naturally occurring allelic variation for some of these pathway steps has been shown to affect variation in carotenoids in a wide variety of maize germplasm. Considering recent advances in our understanding of metabolic pathway reactions potentially involved with variation in carotenoid concentration and composition, it is likely that genes involved upstream of carotenoid synthesis will be prime candidates for the regulation of multiple carotenoid traits, while genes involved within carotenoid synthesis will be candidates for compositional differences. In this study, we investigate the genetic architecture underlying carotenoid concentration and compositional differences in F_2:3_ maize populations A619 × SC55 and DEexp × CI7, two genetic populations derived from high total carotenoid and high β-carotene parent inbreds. We evaluate carotenoid trait profiles and identify QTL across temperate and subtropical environments, map genes of the carotenoid biosynthesis pathway relative to these QTL, and employ a multivariate approach to QTL mapping of carotenoid traits as a possible method to identify major regulatory control points for carotenoid accumulation.

## Materials and methods

### Genetic materials

Two mapping populations were developed from maize inbred lines selected for superior and complementary carotenoid composition and total carotenoid profiles. This information is based on the multi-year surveys of the Goodman-Buckler Diversity Panel (Harjes et al. 2008).

The first population consists of 103 F_2:3_ progeny derived from the cross of DEexp × CI7. DEexp was developed by Dr. Jim Hawk at the University of Delaware by selfing from a southeastern U.S. region Pioneer Hi-bred F_1_ hybrid. CI7, developed by the USDA-ARS, is derived from the backcross L317/33-16//L317. Both lines yield high seed β-carotene concentrations, and also have high total carotenoid concentrations relative to lines screened in the Goodman-Buckler Diversity Panel (Supplemental Figure 1). Specific combining effects for β-carotene have been observed in the DEexp × CI7 hybrid, including high-parent heterosis for β-carotene in some, but not all, years (T. Rocheford, data not shown).

The second population consists of 227 F_2:3_ progeny derived from the cross of A619 × SC55. A619 is an early maturing line developed in Minnesota, derived from the backcross A171/OH43//OH43, and exhibits a high total carotenoid profile relative to most lines in the Goodman Diversity Panels in 2003 and 2005 (top 25 and 20 % respectively, Supplemental Figure 1). SC55 was developed in South Carolina and is characterized by proportionally high β-carotene in grain. Favorable specific combining ability for high β-carotene concentration was observed in hybrid seed of the A619 × SC55 cross (Stevens 2007) and other materials (Egesel et al. 2003).

The above F_1_ crosses were self-pollinated to create the F_2_ generation. The F_2_ seed from a single ear for each cross was planted, and plants were hand self-pollinated to create ears of F_2:3_ derived seed.

### Field evaluation

In 2005, the 103 F_2:3_ lines of DEexp × CI7 and 227 F_2:3_ lines of A619 × SC55 were grown in two replicates in each of two environments, the University of Illinois Urbana-Champaign and the International Maize and Wheat Improvement Center (CIMMYT), El Batan, Mexico (hereafter noted as Illinois environment and Mexico environment, respectively), using alpha (0, 1) incomplete block designs. The families were planted in single row plots of 5 m length, with 76 cm spacing between rows. Each plot was thinned to a density of approximately 15 plants per 5 m, or 43,000 plants ha^−1^. Seven to nine plants were sib-pollinated within each row. Growing seasons varied by environment as follows: Urbana, IL, May–October; El Batan, Mexico, May–November. Fluctuation in average monthly temperature and precipitation recorded at both locations in 2005 is shown in Supplemental Figure 2.

After storage at room temperature for approximately 4 months, seed was bulked for each plot at shelling and an aliquot of approximately 10 g of seed from each plot was stored at −80 °C until carotenoid extraction could be performed on a plot mean basis. Due to field technical complications, the Illinois replicates of population A619 × SC55 were not included this study.

### DNA extraction and genotyping

DNA extraction was performed as described by Mikkilineni and Rocheford (2003). DNA samples prepared from F_2:3_ family seed bulks were used for genotyping. A parental survey using 748 publicly available microsatellite (SSR) markers from MaizeGDB was performed. Pioneer Hi-Bred performed in-kind genotyping on both DEexp × CI7 and A619 × SC55 populations using polymorphic markers identified in the parental survey in addition to several proprietary markers, designated by the prefix “pio”. Additional SSR markers genotyped at the University of Illinois were added to the genetic map. Allele-specific markers *lcy* -MZA, *crtRB1*-InDel4, and *ccd1*-5p were used to map three genes implicated in carotenoid synthesis or degradation: lycopene epsilon cyclase (*lcy;* NM_001153368.1), beta-carotene hydroxylase (*crtRB1;* NM_001112437), and carotenoid cleavage dioxygenase 1 (*ccd1*; DQ100346), respectively. Information for functional marker assays designed for this study (*lcy* and *ccd1*) and those derived from Yan et al. (2010) (*crtRB1*) is listed in Supplemental Table 1.

### Phenotypic data collection

Carotenoid extraction from finely ground maize kernels was performed at Iowa State University as previously described (Li et al. 2007b). Briefly, the method involves mild heat treatment (50 °C × 15 min) in methanol followed by extraction with tetrahydrofuran and a 5-min saponification with methanolic potassium hydroxide at room temperature. After washing with water, the carotenoids are partitioned into hexane/methylene chloride (5:1 v/v), which is then evaporated to dryness under vacuum. The dried extract is reconstituted in methyl-*tert*-butyl ether followed by methanol and an aliquot is injected into the HPLC system. An internal standard, β-apo-8′-carotenal, was added during the extraction. Carotenoid separation was performed on a C-30 YMC Carotenoid Column, with gradient separation (methanol/methyl-*tert*-butyl ether) and photodiode array detection. Carotenoids were quantified using internal standard calibration curves. Measured carotenoid traits included lutein, zeaxanthin, zeinoxanthin, β-cryptoxanthin, α-carotene, *trans* and *cis* isomers of β-carotene, phytoene and *trans*-phytofluene. The summation of all carotenoids with absorption spectra in the visible range (lutein, zeaxanthin, zeinoxanthin, β-cryptoxanthin, α-carotene, total β-carotene) is termed total colored carotenoid in subsequent analyses. Isomers of β-carotene measured in this study include all-*trans*, 9-*cis*, 13-*cis*, and 15-*cis* β-carotene, and are represented by concentration (μg β-carotene g^−1^ DW) and proportion (ratio of isomer to total β-carotene). The ratio of colorless to colored carotenoids is defined by the proportion of phytoene plus phytofluene to total colored carotenoid. Proportion of alpha branch carotenoids (lutein, zeinoxanthin, α-carotene) to beta branch carotenoids (zeaxanthin, β-cryptoxanthin, β-carotene) is defined by α:β branch. Provitamin A was calculated according to the number of retinyl groups associated with all measured carotenoids, resulting in the expression ProVA = β-carotene + 0.5*β-cryptoxanthin + 0.5*α-carotene. All other ratios are comprised of absolute concentrations.

### Genetic map construction

Genetic maps for both populations were constructed using JoinMap^®^ Version 3 (Ooijen and Voorrips 2001). Genotype data were screened for segregation distortion; markers with genotype segregation significantly different from the predicted 1:2:1 Mendelian ratio were removed. Maps were created from individual linkage groups using a LOD threshold of *P* < 0.001 and recombination frequency of 0.49 using Haldane’s map function. The final linkage map for DEexp × CI7 consisted of 109 markers, spaced with an average interval length of 15 cM over a total of 1,486 cM. The final linkage map for A619 × SC55 consisted of 117 markers, spaced with an average interval length of 16.2 cM over a total of 1,728.6 cM. Genetic maps for both populations are shown in Supplemental Figure 3.

### Phenotypic data analyses

All analyses were carried out using statistical procedures in SAS version 9.2 (SAS Institute 2008). Trait means and ranges by environment and for combined environments were obtained through Proc MEANS. Product-moment correlations of raw trait data were performed using Proc CORR. Analysis of variance for trait data was modeled according to $y_{ijkl} = \mu + \alpha_{i} + \beta_{j(i)} + \delta_{k(ij)} + \gamma_{l} + (\alpha \gamma )_{il} + e_{ijkl}$ where *y* is the carotenoid phenotype (μg g^−1^) of an individual in the *i*th environment, *j*th replicate, *k*th block and *l*th genotype, *μ* is the population mean, *α* is the effect of the *i*th environment, *β* is the effect of the *j*th replication in the *i*th environment, *δ* is the effect of the *k*th block in *j*th replication of the *i*th environment, *γ* is the effect of the *l*th genotype (or family), (*αγ*) is the interaction effect of the *i*th environment and the *l*th genotype, and *ε* is the experimental error. The analyses were performed with Proc MIXED and all model effects were considered random. Variance component estimates for genetic variance ($\sigma_{g}^{2}$), genotype by environment interaction variance ($\sigma_{\text{ge}}^{2}$), and error variance ($\sigma_{{}}^{2}$) were performed using Proc VARCOMP. Broad-sense heritability estimates were calculated on an entry mean basis as described by Hallauer and Miranda (1988).

Best linear unbiased predictors (BLUPs) of F_2:3_ family means for all traits were calculated in each population by environment for further use in principal component and linkage analyses. Residuals were checked for normality and independence prior to the calculation of trait BLUPs. Trait data were modeled according to: $y_{ijkl} = \mu + \beta_{j} + \gamma_{l} + e_{jl}$, where *β* is the effect of the *j*th replication, *γ* is the effect of *l*th genotype (or family), and *ε* is the experimental error. Block effects were not included as they were not found to significantly account for phenotypic variation when included in models.

### Principal component analysis

Principal component analysis (PCA) was conducted for each population using Proc PRINCOMP in SASv9.2 using a matrix of trait BLUPs for colored carotenoids (lutein, zeaxanthin, zeinoxanthin, β-cryptoxanthin, α-carotene, β-carotene) plus colorless carotenoids phytoene and phytofluene.

### Linkage analysis by composite interval mapping

Quantitative trait locus analysis was performed using composite interval mapping (CIM) with PLABQTL software (Utz and Melchinger 1993). This mapping methodology is based on the Haley-Knott regression method for marker intervals (Haley and Knott 1992), and is supplemented by cofactor selection (Jansen and Stam 1994; Zeng 1994). Univariate (single trait BLUP values) and multivariate (PC score) traits were mapped according to the following model: $y_{j} = a + B_{i} x_{ij} + \sum\nolimits_{k \ne i} {g_{k} z_{kj} }$ where *y* is the trait value of family *j* (μg g^−1^), *a* is the intercept, *B*_*i*_ is the genetic effect of the QTL located within the interval between marker *i* and marker *i* + 1, *x*_*i*_ is a coded variable indicating the genotype of the marker interval (where maximally the value of parent 1 is −1, and parent 2 is 1), $\sum\nolimits_{k \ne i} {g_{k} z_{kj} }$is the summation of selected cofactor effects where *g*_*k*_ is the partial regression coefficient of the trait value on marker cofactor *k* and *z*_*kj*_ is a coded variable for the genotype at cofactor *k*.

A LOD threshold equivalent to an experiment-wise Type I error rate of *α* = 0.25 was used as the initial model selection criterion (Lander and Botstein 1989) for both populations; to obtain the threshold, 1,000 permutations of the trait data were run using a model of specified gene action, cofactor selection (Doerge and Churchill 1996), and a 2 cM scan. This corresponded to a threshold of LOD 3.5 for DEexp × CI7 analyses and LOD 2.9 for A619 × SC55 analyses. Regression models accounting for digenic additive by additive interactions or dominance effects were tested using the marker set selected by stepwise regression. Model fit criteria including Akaike’s Information Criterion (AIC) and adjusted *R* ^2^ were used to select models accounting for greatest phenotypic variation with fewest estimated parameters. For most traits in both populations, an additive model produced an adequate fit to the data.

Adjusted *R* ^2^ values from the final multiple regression model express the proportion of phenotypic variation adjusted by the number of estimated model parameters. Additive effects are noted for each QTL, and reflect the partial regression coefficient of the marker genotype on the phenotype. These effects are expressed as a deviation from the second inbred parents, which in this study are CI7 and SC55. Effects associated specifically with the *crtRB1* locus have been previously noted in population DEexp × CI7 by Yan et al. (2010).

## Results

### Comparison of descriptive statistics for traits across locations

**Fig. 2:**
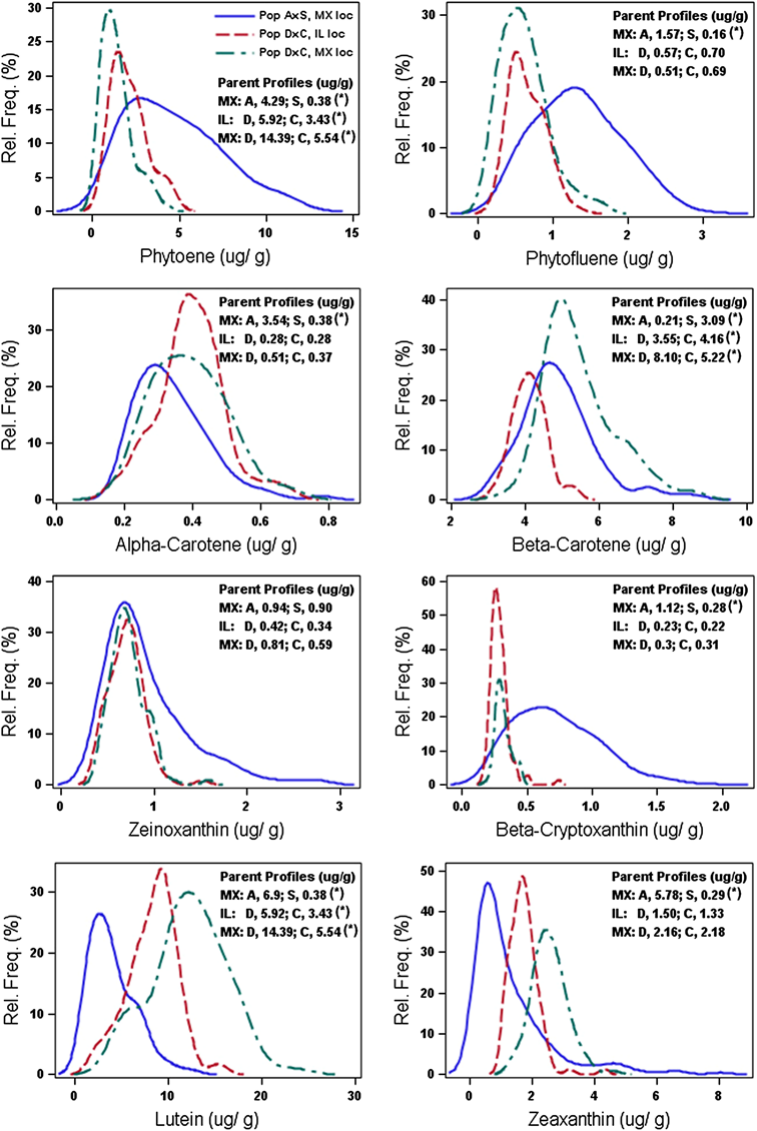
Distribution of measured carotenoid traits by population and environment. Carotenoid concentrations measured for individuals in each population-environment combination are represented by frequency histograms proportional to the size of each population. Data are shown by population-environment for A619 × SC55/MX (*blue solid line*), DEexp × CI7/IL (*red dashed line*), and DEexp × CI7/MX (*green dashed line*), respectively. Parent profiles are listed within respective bar graphs by population-environment and significantly different means (*α* = 0.05) are noted by (*asterisk*) (color figure online)

Eight carotenoid traits (lutein, zeaxanthin, zeinoxanthin, β-cryptoxanthin, α-carotene, β-carotene, phytoene and phytofluene) and four β-carotene isomers (9-*cis*, 13-*cis*, 15-*cis* and all-*trans* β-carotene) were measured in parent inbreds and F_2:3_ progeny for population DEexp × CI7 across two environments and for population A619 × SC55 in one environment (Mexico). Six derived carotenoid traits including total carotenoid concentration, total colored carotenoid concentration, colored:colorless carotenoid ratio, β-carotene conversion (β-car: β-cry ratio), α:β branch conversion (α:β ratio) and provitamin A (ProVA) were calculated from measured carotenoid traits (Supplemental Figure 4). DEexp and CI7 parent inbreds differed significantly (*α* = 0.05) in lutein, β-carotene and phytoene concentrations in both environments (Fig. 2). High total carotenoid concentrations were observed in A619, DEexp and CI7, while SC55 had high β-carotene concentrations relative to other experimental germplasm planted in the same environment year (Supplemental Figure 1), confirming the selection criteria used for these parents.

Heritabilities on an entry mean basis (*h* ^2^) for carotenoid concentrations were high in population DEexp × CI7: phytoene, 0.68; phytofluene, 0.71; α-carotene, 0.71; total β-carotene, 0.59; zeinoxanthin, 0.82; β-cryptoxanthin, 0.79; lutein, 0.74; zeaxanthin, 0.75. Both concentrations (*h* ^2^ = 0.56–0.64) and relative proportions (*h* ^2^ = 0.54–0.74) of the β-carotene isomers were found to be highly heritable. The effect of genotype was significant (*α* = 0.01) for all carotenoids in population DEexp × CI7, and genotype × environment (G×E) interaction effects were significant for lutein, β-carotene, phytoene and phytofluene.

The environment effect was significant (*P* < 0.05) for all traits in the DEexp × CI7 F_2:3_ population except α-carotene. Carotenoid concentrations were larger in the Mexico than in the Illinois environment, with the exception of colorless carotenoids (phytoene and phytofluene) which were smaller in Mexico (Fig. 2, Supplemental Fig 4, Supplemental Fig 5). Trait concentrations and ratios were normally distributed in the DEexp × CI7 F_2:3_ population and transgressive segregation for all traits was observed in both directions for both environments. This contrasted sharply with trait distributions from the A619 × SC55 F_2:3_ population in the Mexico environment, in which distributions for nearly all carotenoids were skewed toward the direction of the SC55 parent profile, and transgressive segregation was observed for all carotenoids at the higher end of trait distributions. Across both populations, *cis* isomers of β-carotene constituted a much smaller proportion of total β-carotene relative to the all-*trans* isomer (50–80 % of total), with relative proportions of: 9-*cis*, 10-25 %; 13-*cis*, 12–20 %; 15-*cis*, 2-6 % (Supplemental Fig 5).

### Correlation and principal component analyses for carotenoid concentrations

Pairwise correlations among carotenoid traits were positive and significant (*α* = 0.05) among all colored carotenoids for the DEexp × CI7 F_2:3_ families (Table 1), except for β-carotene and lutein (*r* = −0.32). Small but significant negative correlations were observed between colorless carotenoids (phytoene and phytofluene) and several colored carotenoids. Correlations among carotenoid concentrations were positive among nearly all colored and colorless carotenoids, and no traits were negatively correlated in the A619 × SC55 population (Table 1).

**Table 1:**
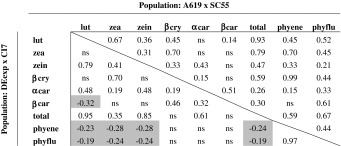
Correlations between kernel carotenoid concentrations within A619 × SC55, and DEexp × CI7 F_2:3_ populations

Significant positive correlations shown in white; significant negative correlations shown in light gray; non-significant correlations are indicated by ns. Significance tested at *a* = 0.05. Traits are coded for lutein (lut), zeaxanthin (zea), zeinoxanthin (zein), β-cryptoxanthin (bcry), α-carotene (acar), β-carotene (bcar), total colored carotenoid (total), phytoene (phyene) and phytofluene (phyflu)

For DEexp × CI7 F_2:3_ progeny, three PCs accounted for 97 % of the total variance in the trait set from the Mexico environment (Table 2). PC1 accounted for the majority of the variance (90 %, *λ* = 8.59) almost entirely through variation in lutein (0.99). PC2 explained 5 % of the trait variance (*λ* = 0.51) through substantial positive loadings in colorless carotenoids (phytoene, 0.91; phytofluene, 0.33) and β-carotene (0.22), whereas PC3 explained a similar amount of variation (4 %, *λ* = 0.36) through positive loadings for lutein (0.23) and β-carotene (0.95), and a negative loading for phytoene (−0.18).

**Table 2:** Principal component parameters for DEexp × CI7 and A619 × SC55 F_2:3_ populations describing carotenoid trait variation

Population	DEexp × CI7			A619 × SC55			
	PC1	PC2	PC3	PC1	PC2	PC3	PC4
Eigenvalues (*λ*)	8.59	0.51	0.36	5.92	4.73	0.80	0.42
Total variance (%)	0.90	0.05	0.04	0.49	0.39	0.07	0.03
Eigenvectors							
Lutein	*0.99*	0.08	0.05	*0.57*	*0.71*	**−0.33**	**−0.25**
Zeaxanthin	0.02	−0.12	*0.23*	*0.26*	*0.31*	*0.75*	*0.49*
Zeinoxanthin	0.04	−0.01	0.05	0.03	0.06	0.02	0.02
β-cryptoxanthin	0.00	−0.01	0.03	0.05	0.04	0.15	0.08
α-carotene	0.01	0.00	0.05	0.01	0.00	−0.04	0.03
β-carotene	−0.07	*0.22*	*0.95*	0.05	0.00	**−0.56**	*0.83*
Phytoene	−0.06	*0.91*	**−0.18**	*0.76*	**−0.63**	0.00	−0.03
Phytofluene	−0.02	*0.33*	−0.07	0.15	−0.09	0.04	−0.05

Carotenoid concentrations included in principal components derived for colored carotenoids (vis) and all carotenoids (path) are listed. Substantial component loadings >0.15 are highlighted in italics; those <−0.15 are highlighted in bold

Four PCs explained the variation in the carotenoid trait matrix for A619 × SC55, with PC1 and PC2 accounting for 49 % (*λ* = 5.92) and 39 % (*λ* = 4.73) of the trait variance in the Mexico environment, respectively, through substantial loadings in lutein, zeaxanthin and phytoene. PC3 and PC4 accounted for 7 and 3 % of the variation, respectively, and had substantial loadings for lutein, zeaxanthin and β-carotene. Eigenvectors for the identified A619 × SC55 PCs demonstrated both synergistic and antagonistic effects through positive and negative loadings.

Loadings for PC traits were interpreted in the context of the biochemical pathway. In DEexp × CI7, most of the pathway variations were attributed to lutein, whereas smaller trait correlations appeared to be involved in apportioning carotenoid branch precursors to β-carotene (PC2) or could be involved in competition between phytoene precursors and downstream carotenoids (PC3). A619 × SC55 PCs were interpreted as a description of synergistic fluxes (PC1) or competitive allocation (PC2) of phytoene substrate to the largest carotenoid pools (lutein and zeaxanthin). PC3 described a competition between lutein and β-carotene versus zeaxanthin, whereas PC4 described a competition between α and β branch carotenoids.

### Quantitative trait locus mapping in DEexp × CI7 population

For the DEexp × CI7 population, marker-genotype association with trait BLUPs from Mexico identified from 2 to 8 QTL explaining significant variation for the various carotenoid concentrations and ratios (Table 3, Supplemental Tables 2 and 3). Multiple regression models accounted for 20.3–55.3 % (*R*_adj_^2^) of the phenotypic variation in individual carotenoid concentrations, and 31.8–58.6 % (*R*_adj_^2^) of variation in ratios.

**Table 3:** QTL detected by composite interval mapping for carotenoid composition traits in DEexp × CI7 population, Mexico environment, 2005

Chr. Bin	Contig	Interval	Pos.	LOD	Add	*R* ^2^ (%)	LOD	Add	*R* ^2^ (%)	LOD	Add	*R* ^2^ (%)
				Phytoene		*R*_2_^adj^: 48.4	Phytofluene		*R*_2_^adj^: 27.8			
3.04/05	120/125	umc1683–umc1102	74	11.66	−0.40	20.9	7.68	−0.12	12.6			
3.08	146	umc1273–pio6	142	5.06	0.20	9.6	4.62	0.06	3.7			
4.00		pio7–pio8	0	4.18	−0.22	11.6						
4.03/04	158/164	adh2–umc2061	52	5.91	−0.23	9.8	4.93	−0.14	17.7			
6.00/02	260/271	umc1018–umc1083	46	9.11	−0.30	16.7						
9.07/08	391	bnlg1375–umc1505	112	5.18	0.23	9.3						
10.06	415	CrtRB1–bnlg1028	54	10.75	−0.41	23.0	9.17	−0.13	13.6			

Chr. Bin	Contig	Interval	Pos.	LOD	Add	*R* ^2^ (%)	LOD	Add	*R* ^2^ (%)	LOD	Add	*R* ^2^ (%)
				β-carotene		*R*_2_^adj^: 55.6	β-cryptoxanthin		*R*_2_^adj^: 37.6	Zeaxanthin		*R*_2_^adj^: 20.3
2.02/04	70/75	umc1756–umc1026	42				7.95	−0.02	22.2			
2.06	91	pio4–umc2194	66	4.8	−0.18	8.7				5.13	−0.23	16.2
3.04/05	120/125	umc1683–umc1102	76	4.2	−0.20	9.7						
4.04/05	164/172	umc2061–umc1895	66	3.67	0.15	7.0						
5.03	210/219	umc2060–umc1692	82	6.89	−0.31	18.8						
5.06/09	251/254	umc2198–umc2209	178	3.92	−0.28	18.3						
7.02	298/301	umc2327–phi034	72	8.16	−0.34	24.6						
7.03	318	bnlg1070–pio11	92				4.06	−0.02	10.6			
8.04/07	353/363	umc1343–bnlg1828	80	6.82	0.35	18.4	10.43	0.03	25.6			
9.01	368/371	bnlg2122–umc1588	14	3.68	−0.11	3.3						
10.06	415	CrtRB1–bnlg1028	56							3.79	0.16	8.9
10.06	415	bnlg1028–phi323152	58				4.02	0.01	5.1			

Chr. Bin	Contig	Interval	Pos.	LOD	Add	*R* ^2^ (%)	LOD	Add	*R* ^2^ (%)	LOD	Add	*R* ^2^ (%)
				α-carotene		*R*_2_^adj^: 22.0	Zeinoxanthin		*R*_2_^adj^: 21.7	Lutein		*R*_2_^adj^: 39.5
1.09	57	umc2028–pio3	188				4.06	0.05	5.7			
2.02	70/75	umc1756–umc1026^a^	30				4.47	−0.04	3.7			
2.04	77	umc1465–pio4	64	7.59	−0.02	8.5						
2.06/08	101/103	umc2205–umc1745	112				3.66	−0.05	8.6			
3.04	116/120	umc1025–bnlg1019^a^	68				5.04	0.09	18.7			
3.04	120/123	bnlg1019–umc1683	70							6.24	1.35	12.7
5.03	210/219	umc2060–umc1692	84	5.71	−0.04	15.5						
6.06/07	288/289	umc1296–1897	154							3.89	1.02	5.3
8.04/07	353/363	umc1343–bnlg1828	66							5.23	−3.01	34.1
9.01	368/371	bnlg2122–umc1588	14	6.25	−0.02	4.7						
		Interaction^a^						−0.06	4.3			

Genetic environment, significance and effect of selected QTL for each trait are listed. Indicated are LOD (Logarithm of Odds), Add (Additive effect) and *R* ^2^ (%) (coefficient of partial determination). Significant digenic interactions detected between main effect QTL are indicated at the bottom of the list of main effects; contributing main effects are marked by asterisk(s)

Although DEexp and CI7 carotenoid profiles derived from the Mexico environment were very similar (Fig. 2), significant QTL were detected for several traits in the corresponding F_2:3_ progeny (Table 3, Supplemental Table 2). Variation in concentrations and trait ratios for colorless carotenoids was attributed to 4–8 main effect QTL which predominantly were located on chromosomes 3 and 4. A larger proportion of the favorable additive effects at these QTL were contributed by DEexp relative to CI7. Eight QTL were detected for β-carotene, several of which individually accounted for more than 10 % of the variation for this trait; most of the positive additive effects for these QTL were also derived from DEexp. Variation in lutein, the carotenoid differing most between inbreds DEexp and CI7, was explained by only two large QTL detected at chromosome 3 (pos. 70, positive CI7 effect of 1.19 μg g^−1^) and chromosome 8 (pos. 66, positive DEexp effect of 2.81 μg g^−1^). Epistatic effects were significant in only two derived traits, α:β branch ratio and colorless:colored carotenoid ratio. While it would be expected that metabolic QTL originating from the same pathway would yield more epistatic interactions, it is likely that the sample size of this population prevented detection of such effects.

Fewer carotenoid QTL were detected for the DEexp × CI7 in Illinois than described above for the Mexico environment. This was attributed to larger trait averages and variances in Mexico than Illinois (Fig. 2). A single, large effect QTL on chromosome 8 (pos. 52–54, *R*_part_^2^ = 40.3) explained variation in lutein, and although this QTL did not map to the exact pos. as those accounting for variation in lutein in the Mexico environment (Mexico, pos. 66; Illinois pos. 52–54), the magnitude of additive effect and source of parental contribution of the lutein QTL were nearly identical across the two environments, suggesting that the same locus could be operating in both environments. Similarly, QTL for zeaxanthin, β-cryptoxanthin, total colored, colorless:colored ratio, βcar:βcry ratio, α:β branch ratio accounting for trait variation in the DEexp × CI7 population grown in Illinois were also detected in the Mexico environment, and had consistent parent of origin and genetic effects (Supplemental Table 3, Table 3). No loci were found to account for significant variation for α-carotene, β-carotene, phytoene or phytofluene (Supplemental Table 3) for DEexp × CI7 in the Illinois environment.

QTL included in the final regression models were found to overlap among carotenoid traits known to be related by way of sequence in metabolic pathways (i.e. colorless or β-cyclized carotenoids) or through extent of hydroxylation (i.e. carotenes or xanthophylls). Colorless carotenoids phytoene and phytofluene shared common QTL at chromosomes 3 (pos. 74; pos. 142), 4 (pos. 52), and 10 (pos. 54). β-cyclized carotenoids (β-carotene, β-cryptoxanthin and zeaxanthin) shared common QTL at chromosomes 2 (pos. 66), 8 (pos. 80), and 10 (pos. 56–58). A β-branch relationship was exposed through a chromosome 10 QTL, which increased the β-carotene:β-cryptoxanthin ratio through the DEexp allele (ratio effect = 0.96, *R*_part_^2^ = 26.3 %) yet increased zeaxanthin through the CI7 allele. α-and β-carotenes were both affected by a QTL on chromosome 2 (pos. 64–66) and another on chromosome 5 (pos. 82–84) with both positive effects derived from the DEexp alleles. The most pervasive pleiotropic effects on the entire pathway were detected at chromosome 2 (pos. 32–40) and chromosome 3 (pos. 68–72), which both increased total colored carotenoids in addition to many individual carotenoids.

QTL mapping for PC1, which accounted for 90 % of the variance among the carotenoid trait matrix, revealed two major QTL at chromosomes 3 (pos. 70) and 8 (pos. 66) to account for 37.8 % of the total phenotypic variation at the Mexico environment (Table 5). These effects were similar to those found for lutein in the same environment, and coincided with PC1 QTL detected in the Illinois environment (chromosome 3, pos. 72, *R*_part_^2^ = 14.9; chromosome 8, pos. 66, *R*_part_^2^ = 43.8; Supplemental Table 3).

Variation in β-carotene isomer concentrations and proportions mapped to 3–8 loci for all-*trans*, 9-*cis* and 13-*cis* β-carotene, accounting for 25–60.5 % of the phenotypic variation (Supplemental Table 4). No QTL were significantly associated with 15-*cis* β-carotene in this population. Several QTL accounted for variation in more than one isomer, including loci on chromosomes 5 (pos. 92–98; 13-*cis*, all-*trans*), 7 (pos. 70–76; 9-*cis*, 13-*cis*, all-*trans*), and 10 (pos. 18–52; 9-*cis*, 13-*cis*, all-*trans*). Similarly, overlapping effects for more than one proportion trait were observed for QTL on chromosomes 2 (pos. 77–90; 9-*cis*, all-*trans*), 5 (pos. 94–96; 9-*cis*, all-*trans*), and 8 (pos. 70–80; 9-*cis*, 13-*cis*, all-*trans*).

### Quantitative trait locus mapping in A619 × SC55 population

Trait BLUPs from the Mexico environment were used to identify significant marker genotype-phenotype associations for the A619 × SC55 population. A range of 3–6 QTL was found to explain 21.6–56.2 % of the variation for individual carotenoid concentrations, and 37.2–47.4 % for carotenoid ratios (Table 4, Supplemental Table 5). Fewer additive by additive interactions were detected in the A619 × SC55 population than in DEexp × CI7. A model for total colored carotenoids explained 53.9 % of the phenotypic variation through four main effects and one epistatic interaction. Variation in two traits, β-carotene and β-carotene:β-cryptoxanthin ratio, was more fully explained by models including dominance effects than only additive effects (data not shown).

**Table 4:** QTL detected by composite interval mapping for carotenoid composition traits in A619 × SC55 population, Mexico environment, 2005

Chr. Bin	Contig	Interval	Pos.	LOD	Add	*R* ^2^ (%)	LOD	Add	*R* ^2^ (%)	LOD	Add	*R* ^2^ (%)
				Phytoene		*R*_2_^adj^: 38.1	Phytofluene		*R*_2_^adj^: 48.0			
1.11	64	umc2242–umc1979	178	3.30	−0.50	3.2	3.13	−0.16	7.9			
3.07	142	pio_5–umc1489	134				5.91	−0.16	8.3			
5.06	251	pio_9–umc2013^a^	144				2.91	0.06	1.4			
7.02	297	umc1068–bnlg1094^a^	46	13.10	−1.45	23.8	22.07	−0.34	32.5			
7.04	323/325	umc1944–umc1125	120	4.30	0.80	9.1						
9.08	391	zct128–umc1505	166	6.59	−1.33	19.6	8.07	−0.25	19.6			
		Interaction^a^						−0.11	2.3			

Chr. Bin	Contig	Interval	Pos.	LOD	Add	*R* ^2^ (%)	LOD	Add	*R* ^2^ (%)	LOD	Add	*R* ^2^ (%)
				β-carotene		*R*_2_^adj^: 37.1	β-cryptoxanthin		*R*_2_^adj^: 45.5	Zeaxanthin		*R*_2_^adj^: 56.2
2.04	77	umc1541–pio_4	128				5.63	−0.12	10.1			
2.05	90	pio_4–umc1459^b^	130							6.74	−1.00	33.5
2.07	91/105	bnlg1396–dupssr25	172	5.97	0.34	10.2						
4.02	156	pio_08–phi295450	32	6.26	0.39	13.5						
4.03	158/176	adh2–umc1142	68	4.34	−0.27	6.6						
5.03	212/217	umc2035–umc2295	72	10.07	0.38	17.5						
6.05	285/287	umc1805–umc1859	90	5.03	0.30	10.9						
7.00	293/296	umc1241–umc1068	46				3.24	−0.08	6.7			
9.07	391	umc1675–umc2099^b^	136							4.32	–0.49	11.6
9.08	391	zct128–umc1505	164				4.07	−0.11	12.4	3.25	−0.41	7.4
10.03	400	umc2017–pio_14	62				3.33	−0.06	2.5			
10.05	414	umc1506–CrtR-B1	88				10.20	−0.21	17.6	4.20	−0.46	10.4
10.06	417	CrtR-B1–umc1993	94	6.43	0.37	14.0						
		Interaction^b^									0.79	19.4

Chr. Bin	Contig	Interval	Pos.	LOD	Add	*R* ^2^ (%)	LOD	Add	*R* ^2^ (%)	LOD	Add	*R* ^2^ (%)
				α-carotene		*R*_2_^adj^: 21.6	Zeinoxanthin		*R*_2_^adj^: 27.0	Lutein		*R*_2_^adj^: 53.6
1.03	–	phi339017–pio_1	72				5.75	0.20	14.6			
–	–	pio_7–pio_8	0	6.84	0.03	11.7						
5.02	208/212	umc1587–umc2035^c^	64	6.06	0.03	8.3	9.29	0.20	16.0			
6.05	283	pio_10–umc1114^c^	76				3.85	−0.10	4.5			
8.04	–	umc1343–LCYe	88	5.93	0.03	9.1						
8.05	354	LCYe–umc1340	92							5.21	0.64	10.0
9.07	391	umc1675–umc2099^d^	136							3.77	−0.46	1.4
9.07	391	CCD1–zct128^d^	162				3.57	−0.08	3.3	9.78	−1.74	18.5
		Interaction^c,d^						−0.1	3.5		0.7	2.2

Genetic environment, significance and effect of selected QTL for each trait are listed. Indicated are LOD (Logarithm of Odds), Add (Additive effect) and *R* ^2^ (%) (coefficient of partial determination). Significant digenic interactions detected between main effect QTL are indicated at the bottom of the list of main effects; contributing main effects are marked by asterisk(s)

Since parent inbred profiles between A619 and SC55 differed for almost all carotenoids, significant QTL were examined for pleiotropic effects. A major effect located on chromosome 9 (pos. 162–166) significantly affected phytoene (1.33 μg g^−1^, *R*_part_^2^ = 19.6 %), phytofluene (0.25 μg g^−1^, *R*_part_^2^ = 19.6 %), zeinoxanthin (0.08 μg g^−1^, *R*_part_^2^ = 3.3 %), lutein (1.74 μg g^−1^, *R*_part_^2^ = 18.5 %), β-cryptoxanthin (0.21 μg g^−1^, *R*_part_^2^ = 17.6 %) and zeaxanthin (0.46 μg g^−1^, *R*_part_^2^ = 10.4 %). Reduction in each of these carotenoid traits was associated with the SC55 allele, which agreed with the low carotenoid profile of inbred SC55.

Pleiotropic effects were observed within pathway branches. A major effect QTL on chromosome 7 (pos. 46) was found to explain 23.8 % of the variation in phytoene and 32.5 % of the variation in phytofluene, with the A619 allele associated with higher levels. A QTL at chromosome 5 (pos. 70–76) augmented total colored carotenoids (1.00 μg g^−1^, *R*_part_^2^ = 8.1 %) and β-carotene (0.38 μg g^−1^, *R*_part_^2^ = 17.5 %) through the SC55 allele, and also decreased colorless:colored carotenoids through the same allele (0.12 μg g^−1^, *R*_part_^2^ = 7.3 %). Competition within the β-branch appeared to be mediated by a QTL on chromosome 10 (pos. 88–94), associated with increased β-carotene (0.37 μg g^−1^, *R*_part_^2^ = 14.0 %) and β-carotene: β-cryptoxanthin (ratio effect = 4.57, *R*_part_^2^ = 40 %), and decreased β-cryptoxanthin (0.21 μg g^−1^, *R*_part_^2^ = 17.6 %) and zeaxanthin (0.46 μg g^−1^, *R*_part_^2^ = 10.4 %) through the SC55 allele. α-branch carotenoids were significantly affected by a QTL located on chromosome 8 (pos. 88–92), which accounted for significant variation in α-carotene, lutein and α/β branch ratio.

Use of allele-specific markers for specific carotenoid biosynthesis genes permitted the comparison of gene map locations with detected QTL effects in this population. *Lycopene epsilon cyclase (lcy)* was mapped to chromosome 8, 92 cM, coinciding with trait effects for several α-branch traits including α-carotene (pos. 88), lutein (pos. 88) and α/β branch ratio (pos. 92). *Beta*-*carotene hydroxylase* (*crtRB1*) mapped to chromosome 10, 93 cM, close to QTL effects for β-cryptoxanthin (pos. 88), zeaxanthin (pos. 88), β-carotene: β-cryptoxanthin (pos. 88) and β-carotene (pos. 94). *Carotenoid cleavage dioxygenase 1* (*ccd1*) mapped to chromosome 9, 145 cM, between two clusters of QTL at pos. 136 (affecting lutein and zeaxanthin) and pos. 162–166 (affecting phytoene, phytofluene, β-cryptoxanthin, zeaxanthin, zeinoxanthin, lutein and total carotenoids).

Genetic effects accounting for variation in four multivariate traits (PCs) were mapped in A619 × SC55. QTL associated with variation in all PC traits consisted of models with 4–5 main effect QTL and few epistatic interactions (Table 5). Models for multivariate traits were found to account for more phenotypic variation (range 31.1–60.4 %) than the univariate carotenoid traits (21.6–56.2 %). Variation in PC1, consisting of significant and positive loadings for phytoene, lutein and zeaxanthin, was explained by five QTL with positive effects predominantly originating from the A619 genome. The QTL at chromosome 9 (pos. 166) with large, pleiotropic effects were observed to affect this PC trait. The PC2 trait, describing antagonism between colorless and colored carotenoids was explained by five QTL, including one observed to have pleiotropic effects on β-branch carotenoids in univariate analysis at chromosome 5 (pos. 76). The PC3 trait, which describes antagonistic variation between zeaxanthin with lutein and β-carotene, was affected by four QTL, one which mapped in close proximity to the *crtRB1* locus on chromosome 10, and another which affected β-branch traits on chromosome 2 (pos. 128). Competition between α- and β-branches, described by PC4, was affected by five main effect QTL and two digenic interactions. Positive effects at all of these QTL were small, and were contributed by SC55 (Table 5). Univariate and multivariate traits overlapped extensively, as shown in Supplemental Figures 6 and 7.

**Table 5:** QTL detected by composite interval mapping for carotenoid principal components in DEexp × CI7 and A619 × SC55 population, Mexico environment, 2005

Pop.	Trait	Model *R*_adj_^2^	Chr. Bin	Contig	Interval	Pos.	LOD	Add	*R* ^2^ (%)
D × C	PC1	37.8	3.04	116/120	bnlg1019–umc1683	70	6.19	1.20	10.2
			8.04/07	353/363	umc1343–bnlg1828	66	4.05	−2.83	31.2
	PC2	52.1	1.03/04	12/14	phi109275–umc2217	100	3.52	−0.22	8.3
			2.00/02	68/69	umc2246–bnlg1017	0	3.74	−0.23	9.6
			3.01	111	umc1892–umc1814	28	7.47	0.31	13.5
			3.04/3.05	120/125	umc1683–umc1102	74	3.99	−0.64	24.7
			3.05	131	umc2265–pio5	90	3.97	0.40	11.3
			3.08	146	umc1273–pio6	142	10.07	0.25	14.5
			4.03/04	158/164	adh2–umc2061	50	4.92	−0.29	16.0
			6.00/02	260/271	umc1018–umc1083	40	9.24	−0.28	18.1
			7.02	298	pio9–umc2327	66	4.53	−0.13	3.3
			9.07/08	391	bnlg1375–umc1505	112	4.34	0.28	12.8
			10.06	415	CrtRB1–bnlg1028	54	15.62	−0.53	34.2
	PC3	68.0	2.00/02	68/69	umc2246–bnlg1017	0	3.78	−0.18	11.2
			2.04	77	umc1465–pio4	64	9.14	−0.30	29.5
			4.06	172/181	umc1895–umc2027	68	4.33	0.22	19.7
			5.03	210/219	umc2060–umc1692	82	12.54	−0.42	38.8
			5.06/09	251/254	umc2198–umc2209	178	4.53	−0.25	22.0
			7.02	298/301	umc2327–phi034	74	8.60	−0.33	31.7
			8.07/09	363/366	bnlg1828–umc1663	94	3.56	0.25	15.1
			9.04/9.05	385	umc1107–umc1094	52	3.76	0.18	10.6
			10.02/03	392/397	umc1576–umc1367	22	4.10	−0.25	16.3
A × S	PC1	60.4	1.11	64	umc2242–umc1979	182	5.18	−0.55	4.8
			7.00	293–296	umc1241–umc1068	44	14.02	−1.30	24.3
			7.04	323/325	umc1944–umc1125	122	2.93	0.49	4.8
			9.07	391	umc1675–umc2099	138	3.81	−0.95	11.9
			9.08	391	zct128–umc1505	166	11.04	−1.63	23.7
	PC2	31.1	–	–	pio_7–pio_8	6	5.79	0.63	5.1
			5.03	212/217	umc2295–bnlg1892	76	4.15	0.85	9.4
			7.04	323	pio_11–umc1944	118	4.06	−0.63	5.8
			8.02	329/345	umc1034–phi115	64	3.36	0.70	6.6
			9.07	391	pio_13–umc1675	116	3.95	−1.43	20.4
	PC3	40.9	2.02	70	umc1934–zca381	88	3.29	−0.29	6.6
			2.04	77	umc1541–pio_4*	128	8.94	−0.68	23.3
			4.08	188/192	umc2187–umc1559*	140	3.07	0.24	4.9
			10.06	417	CrtR-B1–umc1993	94	5.32	−0.37	10.3
					Interaction*			−0.31	3.1
	PC4	36.1	2.08	108	dupssr25–umc1736*	184	4.51	0.2	4.2
			4.02	156	pio_08–phi295450**	28	4.78	0.2	6.8
			5.03	212/217	umc2035–umc2295*	72	8.02	0.3	16.2
			6.02	271/276	umc1178–umc1918**	34	4.23	0.2	4.6
			6.05	285/287	umc1805–umc1859	90	6.01	0.3	11
					Interaction*			0.3	5.9
					Interaction**			0.2	2.9

Genetic environment, significance and effect of selected QTL for each trait are listed. Indicated are LOD (Logarithm of Odds), Add (Additive effect) and *R* ^2^ (%) (coefficient of partial determination)

A range of 3–7 loci accounted for 8.2–36.5 % of the variation in β-carotene isomer concentration and proportion traits in population A619 × SC55 (Supplemental Table 6). As observed in population DEexp × CI7, several loci cosegregated with variation in more than one isomer concentration including: chromosome 5 (pos 72, 13-*cis* and all-*trans*), 10 (pos 92, *CrtRB1* marker; 13-*cis* and all-*trans*). Loci with pleiotropic effects on β-carotene isomer proportion were identified on chromosomes 1 (pos 12; 9-*cis*, all-*trans*), 4 (pos 186; 9-*cis*, all-*trans*), 5 (pos 72; 9-*cis*, 15-*cis*, all-*trans*) and 6 (pos 34-38; 9-*cis*, 15-*cis*, all-*trans*).

### Comparison of detected QTL to known biosynthesis genes

**Fig. 3:**
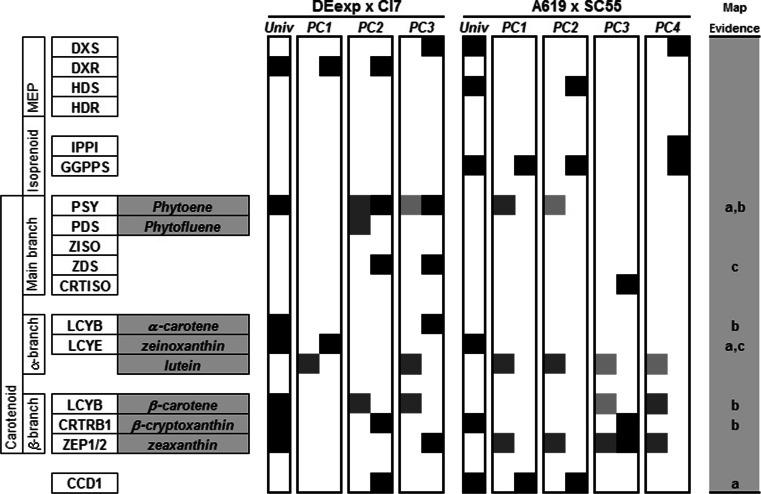
Network of metabolic QTL influencing grain carotenoid concentrations in populations DEexp × CI7 and A619 × SC55. QTL are shown for univariate (single carotenoid concentration) and multivariate (PC) traits mapping to locations of genes known to be in the carotenoid or precursor pathways (overlap delimited by *black squares*). Correlated trait effects are shown for all PC traits, where *colored squares* indicate the carotenoids which are best described by a given PC; the presence of only blue squares shows that these carotenoid variation described by a single PC covaries proportionally, whereas presence of *blue and red squares* indicates an inverse covariation between the carotenoids described by that PC (color figure online)

A total of 63 main effect QTL for carotenoid univariate traits were found to span 29 marker intervals for the DEexp × CI7 F_2:3_ population in the Mexico environment, in contrast to 22 unique main effects for the multivariate principal component traits. For the A619 × SC55 population, a total of 50 main effect QTL across 29 marker intervals were found to explain phenotypic variation in univariate traits, as compared to 19 main effect QTL for multivariate traits across 18 marker intervals. The map locations for univariate and PC traits often overlapped, but QTL that were unique to PC traits were detected in both populations (Supplemental Figure 6 and 7). Many of the detected QTL clusters were found within proximity of mapped carotenoid biosynthesis genes (Andorf et al. 2010; Supplemental Table 6). Although genetic maps for both populations did not provide sufficient resolution to definitively conclude that the known genes and detected QTL mapped within proximity to the same environment (as gauged by chromosome bin assignment), inferences for these gene-phenotype relationships were strengthened by comparing the affected traits with the known metabolic functions of previously identified genes, overlapping detection between univariate and multivariate traits, and detection in previous mapping studies (Fig. 3).

Overall substrate increases to the carotenoid pathway in population DEexp × CI7 were described by PC2, which mapped to chromosome bins also containing genes involved in carbon substrate allocation to the MEP pathway (DXR, bin 3.04) and isoprenoid substrate allocation to the carotenoid pathway (PSY1, bin 6.01). PC3 described the balance between colorless carotenoids and colored pathway intermediates, and mapped near genes involved with carotenoid conversion within the main branch (ZDS, bin 7.02) in addition to conversion of main branch carotenoids to α and β branch carotenoids (LCYβ, bin 5.04). PSY2, an isoform of phytoene synthase in bin 8.07, was also found to map near PC3 QTL. The largest trait variation was observed for lutein, and was represented by the PC1 trait. This PC trait mapped to chromosome bins containing DXR (bin 3.04) and LCYε (bin 8.05), both of which are involved in carotenoid synthesis by committing carbon substrate to the carotenoid pathway, and to the α-branch, respectively.

Multivariate traits for A619 × SC55 also described overall substrate increases to the pathway through PC1 and allocation between colorless substrate and colored intermediates through PC2. These traits mapped close to genes that are involved in the allocation of substrate precursors to the carotenoid pathway (HDS, bin 5.03; GGPPS1, bin 7.04) as well as one that removes carotenoids from the pathway through degradation (CCD1/*wc1*, bin 9.07). Balance between the accumulation of β-carotene and zeaxanthin, explained by PC3, had associated QTL mapping proximal to genes directly involved in zeaxanthin metabolism including crtRB1(bin 10.06) and ZEP1(bin 2.04) as well as carotenoid conversion by CrtISO1 (bin 4.08). PC4 described competition between the α- and β-pathway branches; although QTL for this trait were found to map within the same chromosome bins containing genes allocating substrate to the entire pathway (GGPPS1, bin 2.08; DXS1, bin 6.05).

## Discussion

### Inferences to metabolic functions for detected QTL

The genetic architecture for high β-carotene and total carotenoids as well as various individual and compositional traits was investigated in two F_2:3_ populations, DEexp × CI7 and A619 × SC55. Through map location comparisons of the detected QTL with those reported for genes of the MEP, isoprenoid, and carotenoid pathways, we concluded that a large proportion of the effects detected in this study are likely derived from metabolic QTL. Confidence in the association of known biosynthesis genes with detected QTL was highest when the trait affected by the QTL was known to be a product or substrate of the associated biosynthesis gene (Fig. 3). Major effect QTL previously identified in other genetic mapping studies also support the association between variation in specific carotenoid traits with locations of putative metabolic QTL (Fig. 3; Islam 2004; Wong et al. 2004; Chander et al. 2008; Harjes et al. 2008). Metabolic function has been confirmed by association analyses for several of these loci including *lcy* (Harjes et al. 2008), *crtRB1* (Yan et al. 2010) and CCD1 (Kandianis and Rocheford, data not shown). We emphasize, however, that inferences should be confirmed through additional genetic analyses utilizing functional markers that are in complete and near complete linkage disequilibrium with the alleles associated with trait variation (Andersen and Lubberstedt 2003), and/or through the use of genetic mapping with high density, high throughput markers.

Use of trait correlations in QTL analyses has been reported to result in increased power of QTL resolution, increased precision of parameter estimates, and the simplification of simultaneous hypothesis testing for multiple traits (Gilbert and Le Roy 2004). Multitrait mapping approaches using multivariate techniques (Korol et al. 1995) or transformation of correlated traits to canonical variables which are then used univariately (Mangin et al. 1998) have also been successfully employed. As information describing the size and system of effects from pleiotropic QTL could be lost through the estimation of single trait effects (Jiang and Zeng 1995), there was reason to attempt QTL discovery in this system of correlated traits by pairing univariate and multivariate analyses. Principal component (PC) traits identified using the variance–covariance matrix for eight carotenoids were mapped by composite interval analysis, leading to the detection of 2–8 QTL for each principal component. Using this strategy, we were able to identify loci simultaneously affecting multiple univariate carotenoid traits, which was suggestive of a pleiotropic QTL. As such, functional associations for the detected suite of QTL suggest that carotenoid concentrations are heavily influenced by (1) the allocation of carbon substrate to the carotenoid pathway, and (2) removal of carotenoids from the pathway through CCD1-facilitated degradation or ZEP1-mediated conversion. In contrast, β-carotene concentrations are largely attributed to (1) the allocation of carotenoid substrate from the main branch supplied by phytoene through the bifurcation point at lycopene, (2) competition between α- and β-branches and (3) conversion within the β-branch.

Cross population comparisons highlight differences in some of the genetic variation controlling total colored carotenoid concentrations. Population DEexp × CI7 was influenced by the effects of genetic variation in metabolic steps providing substrate precursors to the carotenoid pathway, including those within the MEP pathway associated with DXS and DXR (Estevez et al. 2001; Rodriguez-Concepcion 2006), as well as reactions that alter allocation within the carotenoid pathway including LCYε. PSY1, encoding phytoene synthase which represents the first committed step of the carotenoid pathway (Buckner et al. 1996), mapped near a QTL accounting for variation in phytoene, which provides the precursor substrate of the major colored carotenoids in grain. A large interaction between chromosomes 2 and 3 affecting the total carotenoid trait may have originated from precursor synthesis at DXR and removal of zeaxanthin through ZEP (Vallabhaneni and Wurtzel 2009). These single trait QTL were confirmed in multitrait analyses, in which PC traits detected genetic variation describing control of overall flux to the pathway (as with DXS and DXR), or allocation of substrate to competing pathway branches (as with LCYε and ZEP). Phenotypic variation for total colored carotenoids within the A619 × SC55 population, on the other hand, was dominated by a large effect proximal to the CCD1 locus. Considering the origin of the deleterious effect came from the SC55 allele, this may explain why skewed trait distributions were observed for nearly all carotenoids in the F_2:3_ progeny of this cross. Interestingly, multitrait analyses for this population highlighted the potential importance of variation in precursor pathways as compared to minimal detection of QTL associated with biosynthesis pathways upstream of the carotenoid pathway in single trait analyses.

Future testing of biosynthesis genes hypothesized to underlie the QTL detected in this (and other) study will likely conclude that few non-metabolic QTL also affect carotenoid trait variation. Some examples of non-metabolic effects modifying carotenoid concentration and composition have been noted in other plant species; for example, reports of mutations in DNA binding domain proteins in cauliflower (Lopez et al. 2008) and plastid development in tomato (Galpaz et al. 2008) demonstrate that regulatory and developmental factors can also control carotenoid biochemistry in developing curd and fruit, respectively. With available maize genomic sequence, it should be possible to search for maize homologs of these genes for further hypothesis testing.

### Modifying carotenoid profiles to address ProVA bioavailability

Much of the allure in modifying metabolic flux through the carotenoid biosynthesis pathway in various crops has been driven by the potential to form provitamin A, and particularly β-carotene (Harjes et al. 2008; Welsch et al. 2010; Yan et al. 2010; Ye and Beyer 2000; Babu et al. 2013). As not all proVA carotenoids are equally absorbed and converted to vitamin A (Tanumihardjo et al. 2010; Davis et al. 2008), proVA bioavailability has emerged as a nutritionally valued trait of similar importance to total proVA concentration.

This study describes natural variation in the β-carotene isomers of maize grain, proVA carotenoids which have been shown to differ in bioavailability. β-carotene in foods is often reported as the compilation of one or more β-carotene isomers, including the most common all-*trans* form, and the less prevalent but more oil-soluble 9-, 13- and 15-*cis* isomers (Schieber and Carle 2005). *Cis*-isomerization of β-carotene is noted to be an artifact of food processing (Achir et al. 2011; Mordi et al. 1993), but also naturally occurs to varying degrees in foods (Khoo et al. 2011). The isomeric state of dietary β-carotene is nutritionally important as its conformation considerably influences its bioavailability. *Trans* isomers of β-carotene are preferentially absorbed (Deming et al. 2002; Stahl et al. 1995). Once absorbed, the 9-*cis* isomer of β-carotene is primarily converted to all-*trans* forms of vitamin A (Maeda et al. 2011). Regarding provitamin A activities, however, 13-*cis* and 9-*cis* β-carotene only maintain 53 and 38 % of the activity conferred by *trans* β-carotene, respectively (Schieber and Carle 2005).

Results from this study reveal a genetic basis for β-carotene isomerization in maize grain, specifically for traits of concentration and proportion of 9-*cis*, 13-*cis*, 15-*cis* and all-*trans* β-carotene. As of yet, there is no evidence for enzymatic conversion of *trans* β-carotene or other carotenoid precursors to *cis* β-carotene. Considering that carotenoids are antioxidants, it is possible that isomerization of *trans* β-carotene under light, heat or oxidative stress could give rise to sizable amounts of *cis* β-carotene. It is also possible that β-carotene isomerization could indeed be facilitated by an isomerase, similar to those which facilitate isomerization of pro-lycopene carotenoids (Chen et al. 2010; Isaacson et al. 2004). This implies that genetic factors influencing *cis* β-carotene accumulation could include: (1) metabolic QTL affecting synthesis or degradation of *trans* β-carotene; (2) as of yet unknown metabolic QTL specific to the formation of *cis* β-carotene and; (3) factors conferring molecular stability of *trans* β-carotene which could influence the degree to which non-enzymatic *trans*–*cis* isomerization occurs, perhaps with changing cellular conditions. Accordingly, we found several QTL with pleiotropic effects for more than one isomer trait to colocalize with known metabolic candidates including ZEP1 (Chr. 2), LCYβ (Chr. 5), LCYε (Chr. 8), and CrtRB1 (Chr. 10).

### Recommendations for future carotenoid breeding studies

The QTL results presented here are consistent with the concept that modification of grain carotenoid profiles primarily originates from variation in the carotenoid biosynthesis pathway, or metabolism that directly creates and allocates substrate to the carotenoid pathway. Association mapping of sequence variation in candidate genes from the carotenoid biosynthesis pathway has revealed that carotenoid composition is significantly altered by genes regulating α- and β-branch allocation through *lcyε* and β-branch conversion through *crtRB1*. From the associations of mapped QTL effects with map locations of known biosynthesis genes, there is reason to expect that increased total carotenoids can be achieved through altered metabolism, and that biochemical reactions upstream of the carotenoid pathway must be targeted to attain this increase. Characterizing allelic variation representative of MEP and isoprenoid pathways through loci such as DXR should be highly informative and useful in association analysis tests and marker-assisted selection studies for total carotenoid grain profiles. Some of the genes in these pathways were detected by PCs representing significant and positive loadings of the major colored carotenoids and phytoene; however, in the case where phytoene and colored carotenoids negatively covary, it may be necessary to include measurement of colorless carotenoids (phytoene and phytofluene) for further genetic analyses.

Factors contributing to efflux from the carotenoid pathway also appear to affect total carotenoid accumulation, through select carotenoids on a specific pathway branch (ZEP) or through non-specific reduction of nearly all carotenoids (CCD1). Compositional differences have been achieved through modification of intra-pathway steps including cyclization reactions (LCYε, LCYβ) and hydroxylation reactions (CRTRB1). Given that most of the phenotypic variation for grain carotenoids in maize exists in lutein, zeaxanthin, zeinoxanthin, β-cryptoxanthin and β-carotene, the steps identified here primarily affect these intermediates and will likely account for most of the phenotypic variation upon selection of the desired alleles. Marker-assisted selection strategies for β-carotene and provitamin A concentrations are already employing variation at *lcyε* and *crtRB1* to attain desired carotenoid profiles. Given the results of this study, there is merit to further investigate the ZEP locus and *lcyβ* for compositional modification, and to evaluate the influence of environment on the metabolic activity of these pathway enzymes.

## Electronic supplementary material

Below is the link to the electronic supplementary material.

Supplemental MAterial 1 (Tables) (DOCX 62 kb)

Supplemental Material 2 (JPG 108 kb)

Supplemental Material 3 (JPG 99 kb)

Supplemental Material 4 (JPG 505 kb)

Supplemental Material 5 (JPG 156 kb)

Supplemental Material 6 (JPG 105 kb)

Supplemental Material 7 (JPG 217 kb)

Supplemental Material 8 (JPG 213 kb)

## References

[CR1] Achir N, Pénicaud C, Avallone S, Bohuon P (2011). Insight into β-carotene thermal degradation in oils with multiresponse modeling. JAOCS.

[CR2] Andersen JR, Lubberstedt T (2003). Functional markers in plants. Trends Plant Sci.

[CR3] Andorf CM, Lawrence CJ, Harper LC, Schaeffer ML, Campbell DA, Sen TZ (2010) The Locus Lookup tool at MaizeGDB: Identification of genomic regions in maize by integrating sequence information with physical and genetic maps. Bioinformatics 26(3):434–436. Art No btp556 10.1093/bioinformatics/btp556 20124413

[CR4] Auldridge ME, Block A, Vogel JT, Dabney-Smith C, Mila I, Bouzayen M, Magallanes-Lundback M, DellaPenna D, McCarty DR, Klee HJ (2006). Characterization of three members of the Arabidopsis carotenoid cleavage dioxygenase family demonstrates the divergent roles of this multifunctional enzyme family. Plant J.

[CR5] Babu R, Rojas NP, Gao S, Yan J, Pixley K (2013). Validation of the effects of molecular marker polymorphisms in LcyE and CrtRB1 on provitamin A concentrations for 26 tropical maize populations. Theor Appl Genet.

[CR6] Bauernfeind JC, Adams CR, Marusich WL (1981). Carotenoids as colorants and vitamin A precursors.

[CR7] Bouis HE, Hotz C, McClafferty B, Meenakshi JV, Pfeiffer WH (2011). Biofortification: a new tool to reduce micronutrient malnutrition. Food Nutr Bull.

[CR8] Buckner B, Kelson TL, Robertson DS (1990). Cloning of the Y1 locus of maize, a gene involved in the biosynthesis of carotenoids. Plant Cell.

[CR9] Buckner B, Miguel PS, JanickBuckner D, Bennetzen JL (1996). The Y1 gene of maize codes for phytoene synthase. Genetics.

[CR10] Chander S, Guo YQ, Yang XH, Zhang J, Lu XQ, Yan JB, Song TM, Rocheford TR, Li JS (2008). Using molecular markers to identify two major loci controlling carotenoid contents in maize grain. Theor Appl Genet.

[CR12] Chandler K, Lipka AE, Owens BF, Li H, Buckler ES, Rocheford T, Gore MA (2013). Genetic analysis of visually scored orange kernel color in maize. Crop Sci.

[CR13] Chen Y, Li F, Wurtzel ET (2010). Isolation and characterization of the Z-ISO gene encoding a missing component of carotenoid biosynthesis in plants. Plant Physiol.

[CR14] Chucair AJ, Rotstein NP, SanGiovanni JP, During A, Chew EY, Politi LE (2007). Lutein and zeaxanthin protect photoreceptors from apoptosis induced by oxidative stress: relation with docosahexaenoic acid. Invest Ophthalmol Vis Sci.

[CR15] Davis C, Jing H, Howe JA, Rocheford T, Tanumihardjo SA (2008). beta-Cryptoxanthin from supplements or carotenoid-enhanced maize maintains liver vitamin A in Mongolian gerbils (*Meriones unguiculatus*) better than or equal to beta-carotene supplements. Br J Nutr.

[CR16] Deming DM, Teixeira SR, Erdman JW (2002). All-*trans* β-carotene appears to be more bioavailable than 9-*cis* or 13-*cis* β-carotene in gerbils given single oral doses of each isomer. J Nutr.

[CR17] Doebley JF, Gaut BS, Smith BD (2006). The molecular genetics of crop domestication. Cell.

[CR18] Doerge RW, Churchill GA (1996). Permutation tests for multiple loci affecting a quantitative character. Genetics.

[CR19] Egesel CO, Wong JC, Lambert RJ, Rocheford TR (2003). Combining ability of maize inbreds for carotenoids and tocopherols. Crop Sci.

[CR20] Estevez JM, Cantero A, Reindl A, Reichler S, Leon P (2001). 1-deoxy-D-xylulose-5-phosphate synthase, a limiting enzyme for plastidic isoprenoid biosynthesis in plants. J Biol Chem.

[CR21] Fierce Y, Vieira MdM, Piantedosi R, Wyss A, Blaner WS, Paik J (2008). In vitro and in vivo characterization of retinoid synthesis from beta-carotene. Arch Biochem Biophys.

[CR23] Galpaz N, Wang Q, Menda N, Zamir D, Hirschberg J (2008). Abscisic acid deficiency in the tomato mutant high-pigment 3 leading to increased plastid number and higher fruit lycopene content. Plant J.

[CR24] Gilbert H, Le Roy P (2004). Power of three multitrait methods for QTL detection in crossbred populations. Genet Sel Evol.

[CR25] Hable WE, Oishi KK, Schumaker KS (1998). Viviparous-5 encodes phytoene desaturase, an enzyme essential for abscisic acid (ABA) accumulation and seed development in maize. Mol Gen Genet.

[CR26] Haley CS, Knott SA (1992). A simple regression method for mapping quantitative trait loci in line crosses using flanking markers. Heredity.

[CR27] Hallauer AR, Miranda JB (1988). Quantitative genetics in maize breeding.

[CR28] Harjes CE, Rocheford TR, Bai L, Brutnell TP, Kandianis CB, Sowinski SG, Stapleton AE, Vallabhaneni R, Williams M, Wurtzel ET, Yan J, Buckler ES (2008). Natural genetic variation in lycopene epsilon cyclase tapped for maize biofortification. Science.

[CR29] Howitt CA, Pogson BJ (2006). Carotenoid accumulation and function in seeds and non-green tissues. Plant Cell Environ.

[CR30] Isaacson T, Ohad I, Beyer P, Hirschberg J (2004). Analysis in vitro of the enzyme CRTISO establishes a poly-*cis*-carotenoid biosynthesis pathway in plants. Plant Physiol.

[CR31] Islam SN (2004) Survey of carotenoid variation and quantitative trait loci mapping for carotenoid and tocopherol variation in maize. Dissertation or Thesis, University of Illinois at Urbana-Champaign

[CR32] Jansen RC, Stam P (1994). High-resolution of quantitative traits into multiple loci via interval mapping. Genetics.

[CR33] Jiang CJ, Zeng ZB (1995). Multiple-trait analysis of genetic-mapping for quantitative trait loci. Genetics.

[CR34] Khoo H-, Prasad KN, Kong K-, Jiang Y, Ismail A (2011). Carotenoids and their isomers: color pigments in fruits and vegetables. Molecules.

[CR35] Korol AB, Ronin YI, Kirzhner VM (1995). Interval mapping of quantitative trait loci employing correlated trait complexes. Genetics.

[CR36] Lander ES, Botstein D (1989). Mapping mendelian factors underlying quantitative traits using RFLP linkage maps. Genetics.

[CR37] Leuenberger MG, Engeloch-Jarret C, Woggon WD (2001). The reaction mechanism of the enzyme-catalyzed central cleavage of beta-carotene to retinal. Angew Chem Int Ed.

[CR38] Li F, Murillo C, Wurtzel ET (2007). Maize Y9 encodes a product essential for 15-*cis*-zeta-carotene isomerization. Plant Physiol.

[CR39] Li S, Tayie FAK, Young MF, Rocheford T, White WS (2007). Retention of provitamin A carotenoids in high β-carotene maize (*Zea mays*) during traditional African household processing. J Agric Food Chem.

[CR41] Li S, Nugroho A, Rocheford T, White WS (2010). Vitamin A equivalence of the β-carotene in β-carotene-biofortified maize porridge consumed by women. Am J Clin Nutr.

[CR42] Lichtenthaler HK, Rohmer M, Schwender J (1997). Two independent biochemical pathways for isopentenyl diphosphate and isoprenoid biosynthesis in higher plants. Physiol Plant.

[CR43] Lois LM, Rodriguez-Concepcion M, Gallego F, Campos N, Boronat A (2000). Carotenoid biosynthesis during tomato fruit development: regulatory role of 1-deoxy-d-xylulose 5-phosphate synthase. Plant J.

[CR44] Lopez AB, Van Eck J, Conlin BJ, Paolillo DJ, O’Neill J, Li L (2008). Effect of the cauliflower Or transgene on carotenoid accumulation and chromoplast formation in transgenic potato tubers. J Exp Bot.

[CR45] Maeda T, Perusek L, Amengual J, Babino D, Palczewski K, Von Lintig J (2011). Dietary 9-*cis*-β, β-carotene fails to rescue vision in mouse models of leber congenital amaurosis. Mol Pharmacol.

[CR46] Mangelsdorf PC, Fraps GS (1931). A direct quantitative relationship between vitamin A in corn and the number of genes for yellow pigmentation. Science.

[CR47] Mangin B, Thoquet P, Grimsley N (1998). Pleiotropic QTL analysis. Biometrics.

[CR48] Matthews PD, Luo RB, Wurtzel ET (2003). Maize phytoene desaturase and zeta-carotene desaturase catalyse a poly-Z desaturation pathway: implications for genetic engineering of carotenoid content among cereal crops. J Exp Bot.

[CR50] Mikkilineni V, Rocheford TR (2003). Sequence variation and genomic organization of fatty acid desaturase-2 (fad2) and fatty acid desaturase-6 (fad6) cDNAs in maize. Theor Appl Genet.

[CR51] Mordi RC, Walton JC, Burton GW, Hughes L, Ingold KU, Lindsay DA, Moffatt DJ (1993). Oxidative degradation of β-carotene and β-Apo-8′-carotenal. Tetrahedron.

[CR52] Ooijen JWV, Voorrips RE (2001). JOINMAP version 3.0, software for the calculation of genetic linkage maps.

[CR53] Palaisa K, Morgante M, Tingey S, Rafalski A (2004). Long-range patterns of diversity and linkage disequilibrium surrounding the maize Y1 gene are indicative of an asymmetric selective sweep. Proc Natl Acad Sci USA.

[CR55] Robertson DS (1975). Survey of albino and white-endosperm mutants of maize: their phenotypes and gene symbols. J Hered.

[CR56] Rodriguez-Concepcion M (2006). Early steps in isoprenoid biosynthesis: multilevel regulation of the supply of common precursors in plant cells. Phytochem Rev.

[CR57] Sandmann G, Roemer S, Fraser PD (2006). Understanding carotenoid metabolism as a necessity for genetic engineering of crop plants. Metab Eng.

[CR58] SAS Institute (2008) SAS/STAT User’s Guide Version 9.2 (SAS Institute, Cary, North Carolina, 2008)

[CR59] Schieber A, Carle R (2005). Occurrence of carotenoid *cis*-isomers in food: technological, analytical, and nutritional implications. Trends Food Sci Technol.

[CR60] Seddon JM, Ajani UA, Sperduto RD, Hiller R, Blair N, Burton TC, Farber MD, Gragoudas ES, Haller J, Miller DT, Yannuzzi LA, Willett W (1994). Dietary carotenoids, vitamin-A, vitamin-C, and vitamin-E, and advanced age-related macular degeneration. JAMA.

[CR61] Singh M, Lewis PE, Hardeman K, Bai L, Rose JKC, Mazourek M, Chomet P, Brutnell TP (2003). Activator mutagenesis of the pink scutellum1/viviparous7 locus of maize. Plant Cell.

[CR62] Stahl W, Schwarz W, Von Laar J, Sies H (1995). All-*trans* β-carotene preferentially accumulates in human chylomicrons and very low density lipoproteins compared with the 9-*cis* geometrical isomer. J Nutr.

[CR63] Stephensen CB (2001). Vitamin A, infection, and immune function. Annu Rev Nutr.

[CR64] Stevens RL (2007) Genetic and QTL analysis of carotenoid variation in two mapping populations of maize. Dissertation or Thesis, University of Illinois at Urbana-Champaign

[CR65] Tang G, Qin J, Dolnikowski GG, Russell RM, Grusak MA (2009). Golden rice is an effective source of vitamin A. Am J Clin Nutr.

[CR66] Tanumihardjo SA, Palacios N, Pixley KV (2010). Provitamin a carotenoid bioavailability: what really matters?. Int J Vitam Nutr Res.

[CR67] Utz HF, Melchinger AE (1993) PLABQTL: a computer program to map QTL 1.2

[CR68] Vallabhaneni R, Wurtzel ET (2009). Timing and biosynthetic potential for carotenoid accumulation in genetically diverse germplasm of maize. Plant Physiol.

[CR69] Vallabhaneni R, Gallagher CE, Licciardello N, Cuttriss AJ, Quinlan RF, Wurtzel ET (2009). Metabolite sorting of a germplasm collection reveals the *Hydroxylase3* locus as a new target for maize provitamin A biofortification. Plant Physiol.

[CR70] Weber EJ (1987). Carotenoids and tocols of corn grain determined by HPLC. J Am Oil Chem Soc.

[CR71] Welsch R, Arango J, Bär C, Salazar B, Al-Babili S, Beltrán J, Chavarriaga P, Ceballos H, Tohme J, Beyera P (2010). Provitamin a accumulation in cassava (*Manihot esculenta*) roots driven by a single nucleotide polymorphism in a phytoene synthase gene. Plant Cell.

[CR72] Whitcher JP, Srinivasan M, Upadhyay MP (2001). Corneal blindness: a global perspective. Bull World Health Organ.

[CR73] Wong JC, Lambert RJ, Wurtzel ET, Rocheford TR (2004). QTL and candidate genes phytoene synthase and zeta-carotene desaturase associated with the accumulation of carotenoids in maize. Theor Appl Genet.

[CR74] Yan J, Kandianis CB, Harjes CE, Bai L, Kim E, Yang X, Skinner DJ, Fu Z, Mitchell S, Li Q, Fernandez MGS, Zaharieva M, Babu R, Fu Y, Palacios N, Li J, DellaPenna D, Brutnell T, Buckler ES, Warburton ML, Rocheford T (2010). Rare genetic variation at *Zea mays* crtRB1 increases beta-carotene in maize grain. Nat Genet.

[CR75] Ye X, Beyer P (2000). Engineering the provitamin A (β-carotene) biosynthetic pathway into (carotenoid-free) rice endosperm. Science.

[CR76] Zeng ZB (1994). Precision mapping of quantitative trait loci. Genetics.

