# Supplementary material for: Genetic architecture controlling variation in grain carotenoid composition and concentrations in two maize populations
Source: Theor Appl Genet. 2013 Sep 17;126(11):2879–95. doi: 10.1007/s00122-013-2179-5 (PMC3825500; doi:10.1007/s00122-013-2179-5)
Supplement: Supplementary file 1 — Supplemental MAterial 1 (Tables) (DOCX 62 kb) [file 122_2013_2179_MOESM1_ESM.docx]

**Supplemental Table 1: Allele-specific functional marker assays used in this study.**

|  |  | ***Allele-Specific Marker Assays*** | | | | |
| --- | --- | --- | --- | --- | --- | --- |
|  |  |  |  |  |  |  |
| ***Gene*** |  | *lcyε* |  | *crtRB1* |  | *ccd1* |
|  |  |  |  |  |  |  |
| ***Accession*** |  | NM_001153368.1 |  | NM_001112437 |  | DQ100346 |
|  |  |  |  |  |  |  |
| ***Marker Assay*** |  | lcye-MZA |  | crtRB1-InDel4 |  | CCD1-pro |
|  |  |  |  |  |  |  |
| ***Assay Type*** |  | PCR, Competitive Binding |  | PCR, InDel |  | PCR, Competitive Binding |
|  |  |  |  |  |  |  |
| ***Polymorphism Targeted*** |  | Exon 1, 3 nt difference btwn A619 & SC55 |  | Exon 1, 12 bp segregating indel |  | Promoter polymorphism |
|  |  |  |  |  |  |  |
| ***Left Primer(s)*** |  | *LYCe-MZA-P1-L (SC55 specific primer)* |  | *crtRB1-D4-F2* |  | *ccd1-WC-L1* |
|  |  | **ATT TTT CTG GTA TTT ATT CAG C** |  | **ACC GTC ACG TGC TTC GTG CC** |  | **CCG TGC TCG GAC AGA ATA GT** |
|  |  |  |  |  |  |  |
|  |  | *LYCe-MZA-P2-L (A619 specific primer)* |  |  |  | *ccd1-B73-rev-L1* |
|  |  | **AAG GCT ACT ACC TCC ATG AAA** |  |  |  | **CTC ACA CGT GTC AAC GCC** |
|  |  |  |  |  |  |  |
| ***Right Primer(s)*** |  | *LYCe-MZA-All-R1* |  | *crtRB1-D4-R1* |  | *ccd1-ALL-R1* |
|  |  | **AAT GAG AAT AGT ATG AGA TCG** |  | **CTT CCG CGC CTC CTT CTC** |  | **GTC GTT TCG GTG GCT GTC** |
|  |  |  |  |  |  |  |
| ***Reference*** |  | this study |  | Yan et al., 2010 |  | this study |
|  |  |  |  |  |  |  |

**Supplemental Table 1**

**Supplemental Table 2: QTL detected by composite interval mapping for derived carotenoid traits in DEexp x CI7, Mexico environment, 2005.** Genetic environment, significance and effect of selected QTL for each trait are listed. Indicated are LOD (Logarithm of Odds), Add (Additive effect) and R^2^ (%) (coefficient of partial determination). Significant digenic interactions detected between main effect QTL are indicated at the bottom of the list of main effects; contributing main effects are marked by asterisk(s).

| Pop. | Trait | Model R^2^_adj_ |  | Chr. Bin | Contig | Interval | Pos. | LOD | Add | R^2^ (%) |
| --- | --- | --- | --- | --- | --- | --- | --- | --- | --- | --- |
|  |  |  |  |  |  |  |  |  |  |  |
| **D x C** | **α: β Branch** | 58.6 |  | 3.04 | 116/120 | bnlg1019 - umc1683 | 70 | 11.33 | 0.19 | 14.8 |
|  |  |  |  | 4.08 | 188 | bnlg2162 - umc2187 ^a^ | 98 | 6.12 | -0.24 | 19.9 |
|  |  |  |  | 5.03/04 | 219/235 | umc1692 - bnlg1208 ^a^ | 100 | 3.73 | 0.04 | 0.5 |
|  |  |  |  | 7.02 | 298/301 | umc2327 - phi034 | 78 | 3.64 | 0.14 | 8.8 |
|  |  |  |  | 8.04/07 | 353/363 | umc1343 - bnlg1828 | 64 | 10.66 | -0.45 | 42.1 |
|  |  |  |  |  |  | interaction ^a^ |  |  | -0.26 | 9.1 |
|  |  |  |  |  |  |  |  |  |  |  |
|  | **β-car: β-cry** | 31.8 |  | 2.02 | 70/75 | umc1756 - umc1026 | 36 | 5.14 | 0.96 | 13.2 |
|  |  |  |  | 10.06 | 415 | CrtRB1 - bnlg1028 | 56 | 9.4 | -1.45 | 26.3 |
|  |  |  |  |  |  |  |  |  |  |  |
|  | **Total Colored** | 35.6 |  | 2.02 | 70/75 | umc1756 - umc1026 | 36 | 5.3 | -1.33 | 10.2 |
|  |  |  |  | 3.04 | 116/120 | bnlg1019 - umc1683 | 70 | 5.06 | 1.04 | 7.8 |
|  |  |  |  | 8.03 | 340 | pio13 - bnlg1863 | 44 | 3.97 | -2.41 | 29.2 |
|  |  |  |  | 9.01/04 | 371 | umc1588 - umc1107 | 16 | 4.61 | -0.88 | 5.4 |
|  |  |  |  |  |  |  |  |  |  |  |
|  | **Colorless: Colored** | 41.6 |  | 3.04/05 | 120/125 | umc1683 - umc1102 | 74 | 9.99 | -0.02 | 13.2 |
|  |  |  |  | 3.08 | 146 | umc1273 - pio6 | 142 | 5.49 | 0.01 | 4.5 |
|  |  |  |  | 4.02/03 | 156/158 | phi295450 - adh2 ^b^ | 48 | 4.52 | -0.02 | 12.9 |
|  |  |  |  | 5.04/05 | 235/239 | bnlg1208 - phi333597 ^b^ | 116 | 3.91 | 0.03 | 14.2 |
|  |  |  |  | 6.00/02 | 260/271 | umc1018 - umc1083 | 46 | 5.75 | -0.01 | 7.0 |
|  |  |  |  | 8.01/02 | 326/329 | umc1483 - umc1034 | 22 | 4.92 | 0.01 | 5.2 |
|  |  |  |  |  |  | interaction ^b^ |  |  | -0.02 | 6.5 |

**Supplemental Table 2**

| Chromosome |  |  | **chr. 2** | | |  | **chr. 3** | | |  | **chr. 5** | | |  | **chr. 8** | | | | | |
| --- | --- | --- | --- | --- | --- | --- | --- | --- | --- | --- | --- | --- | --- | --- | --- | --- | --- | --- | --- | --- |
| Bin |  |  | 2.02-2.04 | | |  | 3.04 | | |  | 5.05 | | |  | 8.01 | | | 8.01-8.02 | | |
| Flanking Markers |  |  | umc1756 - umc1026 | | |  | bnlg1019 - umc1683 | | |  | phi333597 - umc1941 | | |  | bnlg1863 -umc1343 | | | umc1343 - bnlg1828 | | |
| Chr. Position (cM) |  |  | 34-36 | | |  | 72 | | |  | 126 | | |  | 52-54 | | | 66 | | |
| Genetic Parameters | Model R^2^_adj_ |  | LOD | Add. | R^2^ |  | LOD | Add. | R^2^ |  | LOD | Add. | R^2^ |  | LOD | Add. | R^2^ | LOD | Add. | R^2^ |
| Lutein | 38.9 |  |  |  |  |  |  |  |  |  |  |  |  |  | 10.63 | -2.28 | 40.3 |  |  |  |
| Zeaxanthin | 15.7 |  | 4.14 | -0.16 | 18.0 |  |  |  |  |  |  |  |  |  |  |  |  |  |  |  |
| Zeinoxanthin | 20.2 |  |  |  |  |  |  |  |  |  |  |  |  |  | 5.16 | -0.10 | 22.2 |  |  |  |
| β-cryptoxanthin | 15.4 |  | 4.03 | -0.02 | 17.2 |  |  |  |  |  |  |  |  |  |  |  |  |  |  |  |
| α-carotene | * |  |  |  |  |  |  |  |  |  |  |  |  |  |  |  |  |  |  |  |
| β-carotene | * |  |  |  |  |  |  |  |  |  |  |  |  |  |  |  |  |  |  |  |
| Phytoene | * |  |  |  |  |  |  |  |  |  |  |  |  |  |  |  |  |  |  |  |
| Phytofluene | * |  |  |  |  |  |  |  |  |  |  |  |  |  |  |  |  |  |  |  |
| Total colored | 30.4 |  |  |  |  |  |  |  |  |  |  |  |  |  | 8.09 | -2.10 | 32.4 |  |  |  |
| Colorless:colored | 20.3 |  |  |  |  |  |  |  |  |  |  |  |  |  | 5.28 | 0.07 | 22.6 |  |  |  |
| βcar:βcry | 28.2 |  | 7.72 | 1.65 | 31.0 |  |  |  |  |  |  |  |  |  |  |  |  |  |  |  |
| αβ branch ratio | 40.4 |  |  |  |  |  |  |  |  |  |  |  |  |  | 11.1 | -0.46 | 41.6 |  |  |  |
| PC1 | 46.4 |  |  |  |  |  | 3.54 | 1.48 | 14.9 |  |  |  |  |  |  |  |  | 12.4 | -3.05 | 43.8 |
| PC2 | * |  |  |  |  |  |  |  |  |  |  |  |  |  |  |  |  |  |  |  |
| PC3 | 20.9 |  |  |  |  |  |  |  |  |  | 5.58 | -0.39 | 22.5 |  |  |  |  |  |  |  |

**Supplemental Table 3: QTL detected by composite interval mapping for carotenoid composition. traits in DEexp x CI7 population, Illinois environment, 2005.** Stepwise regression models not found to have any significant regression coefficients (QTL) are designated by (*). Genetic environment, significance and effect of selected QTL for each trait are listed. Indicated are LOD (Logarithm of Odds), Add (Additive effect) and R^2^ (%) (coefficient of partial determination).

**Supplemental Table 3**

| Chr. Bin | Contig | Interval | Pos |  | LOD | Add | R^2^ (%) |  |  | LOD | Add | R^2^ (%) |  |  |
| --- | --- | --- | --- | --- | --- | --- | --- | --- | --- | --- | --- | --- | --- | --- |
|  |  |  |  |  | **9-cis BC Cn** | | R_2_^adj^: 25.0 |  |  | **9-cis BC Pr** | | R_2_^adj^: 49.3 |  |  |
| 2.08/09 | 104/108 | phi090-umc1525 | 142 |  | 3.75 | 0.024 | 6.2 |  |  |  |  |  |  |  |
| 7.02 | 298 | pio9 - umc2327 | 70 |  | 4.68 | -0.027 | 9.8 | * |  |  |  |  |  |  |
| 10.06 | 415 | CrtRB1 - bnlg1028 | 52 |  | 4.89 | -0.037 | 15.4 | ** |  |  |  |  |  |  |
|  |  |  |  |  |  |  |  |  |  |  |  |  |  |  |
| 2.04 | 77 | umc1465 - pio4 | 64 |  |  |  |  |  |  | 6.89 | 0.011 | 21.7 | * |  |
| 5.03/04 | 219/235 | umc1692 - bnlg1208 | 96 |  |  |  |  |  |  | 4.44 | 0.012 | 16.8 | ** |  |
| 5.06/09 | 251/254 | umc2198 - umc2209 | 168 |  |  |  |  |  |  | 5.11 | 0.008 | 9.2 |  |  |
| 8.04 | - | umc1343-LCYe | 70 |  |  |  |  |  |  | 5.43 | -0.013 | 16.8 | *** |  |
| 9.01/04 | 371 | umc1588 - umc1107 | 16 |  |  |  |  |  |  | 3.48 | 0.008 | 9.7 |  |  |
|  |  |  |  |  |  |  |  |  |  |  |  |  |  |  |
|  |  |  |  |  | **13-cis BC Cn** | | R_2_^adj^: 41.3 |  |  | **13-cis BC Pr** | | R_2_^adj^: 25.7 |  |  |
| 4.04/05 | 164/172 | umc2061 - umc1895 | 62 |  | 5.18 | 0.022 | 14.3 |  |  |  |  |  |  |  |
| 5.03/04 | 219/235 | umc1692 - bnlg1208 | 98 |  | 6.64 | -0.033 | 21.0 | *** |  |  |  |  |  |  |
| 7.02 | 298/301 | umc2327 - phi034 | 72 |  | 8.36 | -0.030 | 22.7 | * |  |  |  |  |  |  |
| 10.02/03 | 392/397 | umc1576 - umc1367 | 18 |  | 4.11 | -0.025 | 13.3 | ** |  |  |  |  |  |  |
|  |  |  |  |  |  |  |  |  |  |  |  |  |  |  |
| 1 |  | umc1269-umc224 | 20 |  |  |  |  |  |  | 4.81 | -0.003 | 4.1 |  |  |
| 3.04/05 | 120/125 | umc1683 - umc1102 | 78 |  |  |  |  |  |  | 3.3 | 0.004 | 8.4 |  |  |
| 7.03 | 318 | bnlg1070 - pio11 | 94 |  |  |  |  |  |  | 4.55 | 0.004 | 8.2 |  |  |
| 8.04 | - | umc1343-LCYe | 80 |  |  |  |  |  |  | 9.81 | -0.007 | 19.1 | *** |  |
|  |  |  |  |  |  |  |  |  |  |  |  |  |  |  |
|  |  |  |  |  | **all-trans BC Cn** | | R_2_^adj^: 60.5 |  |  | **all-trans BC Pr** | | R_2_^adj^: 46.9 |  |  |
| 2.04 | 77 | umc1465 - pio4 | 64 |  | 6.42 | -0.234 | 18.9 |  |  |  |  |  |  |  |
| 3.04/05 | 120/125 | umc1683 - umc1102 | 78 |  | 3.85 | -0.153 | 8.0 |  |  |  |  |  |  |  |
| 5.03/04 | 219/235 | umc1692 - bnlg1208 | 92 |  | 6.65 | -0.277 | 21.4 | *** |  |  |  |  |  |  |
| 5.06/09 | 251/254 | umc2198 - umc2209 | 178 |  | 5.94 | -0.237 | 18.9 |  |  |  |  |  |  |  |
| 7.02 | 298/301 | umc2327 - phi034 | 76 |  | 7.07 | -0.292 | 25.1 | * |  |  |  |  |  |  |
| 8.04 | - | umc1343-LCYe | 72 |  | 5.32 | 0.261 | 7.0 |  |  |  |  |  |  |  |
| 8.07/09 | 363/366 | bnlg1828 - umc1663 | 94 |  | 5.63 | 0.176 | 3.8 |  |  |  |  |  |  |  |
| 10 | 397/ 400 | umc1367-umc2017 | 28 |  | 4.73 | -0.191 | 11.6 | ** |  |  |  |  |  |  |
|  |  |  |  |  |  |  |  |  |  |  |  |  |  |  |
| 2.05 | 90 | pio_4-umc1459 | 68 |  |  |  |  |  |  | 4.72 | -0.014 | 14.1 | * |  |
| 5.03/04 | 219/235 | umc1692 - bnlg1208 | 94 |  |  |  |  |  |  | 4.79 | -0.018 | 19.8 | ** |  |
| 7 | - | pio11-pio12 | 102 |  |  |  |  |  |  | 3.48 | -0.014 | 11.2 |  |  |
| 8.04 | - | umc1343-LCYe | 74 |  |  |  |  |  |  | 6.32 | 0.026 | 27.8 | *** |  |

**Supplemental Table 4: QTL detected by composite interval mapping for** β**-carotene isomer concentration (Cn) and proportion (Pr) in DEexp x CI7 population, Mexico environment, 2005.** Genetic environment, significance and effect of selected QTL for each trait are listed. Indicated are LOD (Logarithm of Odds), Add (Additive effect) and R^2^ (%) (coefficient of partial determination). QTL common to more than one isomer concentration or proportion are denoted by asterisks.

**Supplemental Table 4**

| Pop. | Trait | Model R^2^_adj_ |  | Chr. Bin | Contig | Interval | Pos. | LOD | Add | R^2^ (%) |
| --- | --- | --- | --- | --- | --- | --- | --- | --- | --- | --- |
|  |  |  |  |  |  |  |  |  |  |  |
| **A x S** | **α: β Branch** | 47.4 |  | 1.01 | 2/4 | umc1177-umc1071 ^c^ | 0 | 2.93 | -0.08 | 2.3 |
|  |  |  |  | 2.05 | 77 | umc1541-pio_4 | 128 | 3.57 | 0.07 | 6.7 |
|  |  |  |  | 6.05 | 283 | pio_10-umc1114 ^c^ | 74 | 5.05 | -0.07 | 0.7 |
|  |  |  |  | 8.05 | 354 | LCYe-umc1340 | 92 | 4.66 | 0.09 | 12.9 |
|  |  |  |  | 9.07 | 391 | CCD1-zct128 | 162 | 10.28 | -0.17 | 31.9 |
|  |  |  |  |  |  | interaction ^c^ |  |  | -0.04 | 0.4 |
|  |  |  |  |  |  |  |  |  |  |  |
|  | **β-car: β-cry** | 39.4 |  | 10.05 | 414 | umc1506-CrtR-B1 | 88 | 10.63 | 4.57 | 40.0 |
|  |  |  |  |  |  |  |  |  |  |  |
|  | **Total Colored** | 53.9 |  | - | - | pio_7-pio_8 | 2 | 3.36 | 0.75 | 4.8 |
|  |  |  |  | 5.03 | 212/217 | umc2035-umc2295 | 70 | 4.43 | 1.00 | 8.1 |
|  |  |  |  | 9.07 | 391 | pio_13-umc1675 | 120 | 6.74 | -1.33 | 7.0 |
|  |  |  |  | 9.07 | 391 | CCD1-zct128 | 162 | 12.76 | -2.32 | 23.9 |
|  |  |  |  |  |  |  |  |  |  |  |
|  | **Colorless: Colored** | 37.2 |  | - |  | pio_7-pio_8 | 2 | 3.71 | -0.08 | 8.0 |
|  |  |  |  | 5.03 | 212/217 | umc2295-bnlg1892 | 76 | 6.09 | -0.12 | 14.7 |
|  |  |  |  | 7.02 | 297 | umc1068-bnlg1094 | 46 | 6.90 | -0.12 | 13.8 |
|  |  |  |  | 7.04 | 323/325 | umc1944-umc1125 | 120 | 3.68 | 0.07 | 5.3 |
|  |  |  |  | 9.07 | 391 | pio_13-umc1675 | 120 | 3.37 | -1.33 | 3.5 |
|  |  |  |  | 10.01 | 392/393 | umc2053-phi059 | 8 | 3.33 | 0.10 | 8.4 |

**Supplemental Table 5: QTL detected by composite interval mapping for derived carotenoid traits in A619 x SC55 population, Mexico environment, 2005.** Genetic environment, significance and effect of selected QTL for each trait are listed. Indicated are LOD (Logarithm of Odds), Add (Additive effect) and R^2^ (%) (coefficient of partial determination). Significant digenic interactions detected between main effect QTL are indicated at the bottom of the list of main effects; contributing main effects are marked by asterisk(s).

**Supplemental Table 5**

| Chr. Bin | Contig | Interval | Pos. |  | LOD | Add | R^2^ (%) |  |  | LOD | Add | R^2^ (%) |  |
| --- | --- | --- | --- | --- | --- | --- | --- | --- | --- | --- | --- | --- | --- |
|  |  |  |  |  | **9-cis BC Cn** | | R_2_^adj^: 19.3 |  |  | **9-cis BC Pr** | | R_2_^adj^: 31.7 |  |
| 1.01 | 4/9 | umc1071-bnlg1953 | 22 |  | 9.16 | -0.072 | 16.5 |  |  |  |  |  |  |
| 6.05 | 285/287 | umc1805-umc1859 | 86 |  | 4.19 | 0.027 | 3.7 |  |  |  |  |  |  |
| 8.02/03 | 345 | phi115-pio12 | 68 |  | 2.67 | 0.019 | 2.0 |  |  |  |  |  |  |
| 9.08 | 391 | zct128-umc1505 | 164 |  | 3.37 | -0.025 | 3.2 |  |  |  |  |  |  |
|  |  |  |  |  |  |  |  |  |  |  |  |  |  |
| 1.01 | 2/4 | umc1177-umc1071 | 12 |  |  |  |  |  |  | 7.66 | -0.015 | 19.4 | * |
| 4.09/ 11 | 163/ 200 | umc1574-phi076 | 186 |  |  |  |  |  |  | 3.11 | -0.006 | 3.5 | ** |
| 5.03 | 212/217 | umc2035-umc2295 | 72 |  |  |  |  |  |  | 3.19 | -0.006 | 4.7 | *** |
| 6.02 | 271/276 | umc1178-umc1918 | 34 |  |  |  |  |  |  | 3.96 | -0.011 | 11.4 | **** |
|  |  |  |  |  |  |  |  |  |  |  |  |  |  |
|  |  |  |  |  | **13-cis BC Cn** | | R_2_^adj^: 23.8 |  |  | **13-cis BC Pr** | | R_2_^adj^: 8.2 |  |
| 5.03 | 212/217 | umc2035-umc2295 | 72 |  | 5.23 | 0.038 | 9.4 | * |  |  |  |  |  |
| 6.05 | 283 | pio_10-umc1114 | 74 |  | 4.87 | 0.041 | 10.1 |  |  |  |  |  |  |
| 8.00 | 345 | phi115-pio12 | 70 |  | 3.18 | 0.028 | 5.4 |  |  |  |  |  |  |
| 10.05 | 414 | umc1506-CrtR-B1 | 92 |  | 4.12 | 0.045 | 9.1 | ** |  |  |  |  |  |
|  |  |  |  |  |  |  |  |  |  |  |  |  |  |
| 3.01/ 02 | 111 | phi104127-umc1886 | 16 |  |  |  |  |  |  | 2.87 | -0.002 | 3.8 |  |
| 3.07 | 142 | pio_5-umc1489 | 134 |  |  |  |  |  |  | 3.59 | 0.004 | 8.6 |  |
| 10.01 | 392/393 | umc2053-phi059 | 44 |  |  |  |  |  |  | 3.27 | -0.001 | 0.4 |  |
|  |  |  |  |  |  |  |  |  |  |  |  |  |  |
|  |  |  |  |  | **15-cis BC Cn** | | R_2_^adj^: 36.5 |  |  | **15-cis BC Pr** | | R_2_^adj^: 30.8 |  |
| 1.03 | 10 | umc2383-bnlg439 | 62 |  | 4.18 | -0.008 | 8.4 |  |  |  |  |  |  |
| 4.00 | - | pio7 - pio8 | 18 |  | 3.69 | 0.006 | 6.4 |  |  |  |  |  |  |
| 6.05 | 285/287 | umc1805-umc1859 | 90 |  | 5.42 | 0.007 | 7.2 |  |  |  |  |  |  |
| 7.02 | 297 | umc1068-bnlg1094 | 48 |  | 4.59 | -0.005 | 3.7 |  |  |  |  |  |  |
| 8 | 345 | phi115-pio12 | 68 |  | 7.08 | 0.006 | 6.3 |  |  |  |  |  |  |
| 9.07 | 391 | umc1675-umc2099 | 130 |  | 4.46 | -0.006 | 7.6 |  |  |  |  |  |  |
| 9.07 | 391 | CCD1-zct128 | 162 |  | 4.04 | -0.006 | 6.2 |  |  |  |  |  |  |
|  |  |  |  |  |  |  |  |  |  |  |  |  |  |
| 2.06 | 91 | pio4 - umc2194 | 130 |  |  |  |  |  |  | 6.08 | -0.003 | 18.4 |  |
| 5.03 | 212/217 | umc2035-umc2295 | 72 |  |  |  |  |  |  | 3.18 | -0.001 | 3.6 | *** |
| 6.02 | 271/276 | umc1178-umc1918 | 38 |  |  |  |  |  |  | 3.62 | -0.002 | 6.1 | **** |
| 10.05 | 414 | umc1506-CrtR-B1 | 92 |  |  |  |  |  |  | 6.08 | -0.003 | 15.8 |  |
|  |  |  |  |  |  |  |  |  |  |  |  |  |  |
|  |  |  |  |  | **all-trans BC Cn** | | R_2_^adj^: 14.4 |  |  | **all-trans BC Pr** | | R_2_^adj^: 33 |  |
| 4.02 | 156 | pio_08-phi295450 | 32 |  | 3.4 | 0.215 | 5.6 |  |  |  |  |  |  |
| 5.03 | 212/217 | umc2035-umc2295 | 72 |  | 6.25 | 0.240 | 9.2 | * |  |  |  |  |  |
| 10.05 | 414 | umc1506-CrtR-B1 | 92 |  | 4.01 | 0.218 | 5.5 | ** |  |  |  |  |  |
|  |  |  |  |  |  |  |  |  |  |  |  |  |  |
| 1.01 | 2/4 | umc1177-umc1071 | 12 |  |  |  |  |  |  | 5.71 | 0.012 | 7.7 | * |
| 1.01 | 4/9 | bnlg1953-umc2383 | 50 |  |  |  |  |  |  | 3.54 | 0.015 | 10.0 |  |
| 4.09/ 11 | 163/ 200 | umc1574-phi076 | 186 |  |  |  |  |  |  | 3.2 | 0.008 | 3.3 | ** |
| 5.03 | 212/217 | umc2035-umc2295 | 72 |  |  |  |  |  |  | 4.46 | 0.007 | 4.0 | *** |
| 6.02 | 271/276 | umc1178-umc1918 | 34 |  |  |  |  |  |  | 6.94 | 0.015 | 12.9 | **** |

**Supplemental Table 6: QTL detected by composite interval mapping for** β**-carotene isomer concentration (Cn) and proportion (Pr) in A619 x SC55 population, Mexico environment, 2005.** Genetic environment, significance and effect of selected QTL for each trait are listed. Indicated are LOD (Logarithm of Odds), Add (Additive effect) and R^2^ (%) (coefficient of partial determination). QTL common to more than one isomer concentration or proportion are denoted by asterisks.

**Supplemental Table 6**

| Pathway Enzyme | Chr. Location | |  |  |  | Traits affected by QTL Mapped to Region | |
| --- | --- | --- | --- | --- | --- | --- | --- |
|  | Bin | Contig | Reference | Mutants | Genbank Acc. | DEexp x CI7 F_2:3_ | A619 x SC55 F_2:3_ |
| **PDS** | 1.02 | 8 | Hable et al. 1998, Li et al. 2007a | *vp5* | NM_001111911.1 |  |  |
| **HDR/ HMDR1** | 1.10 | 57 | † |  | DR789385.1 | zeino |  |
| **ZEP1** | 2.04 | 78 | † |  | DR820114.1 | PC3, βcry, αcar (*) | PC3, βcry |
| **GGPPS1** | 2.08 | 105 | † |  | EF417573.1 |  | PC4, βcar |
| **CrtISO2** | 2.09 | 108 | † |  | DR812825 |  |  |
| **DXR** | 3.04 | 117 | † |  | AJ297566.1 | PC1, PC2, phytoene, phytoflu, βcar, zeino, lut (**) |  |
| **CrtISO1** | 4.08 | 188 | † |  | DR812825 |  | PC3 |
| **LCYb** | 5.04 | 225 | Singh et al. 2003 | *ps1/ vp7* |  | PC3, αcar, βcar |  |
| **HDS/ HMDS** | 5.05 | 247 | † |  | AY562489 |  | PC2, βcar, αcar, zeino |
| **PSY1** | 6.01 | 270 | Buckner et al. 1990, 1996 | *y1* |  | PC2, phytoene |  |
| **IPPI3** | 6.05 | 287 | † |  | CO530856.1 |  | PC4, βcar, zeino |
| **DXS1** | 6.05 | 285 | † |  | NM_001164333.1 |  |  |
| **ZDS** | 7.02 | 297 | Matthews et al. 2003 | *vp9* |  | PC2, PC3, βcar | phytoene, phytoflu |
| **DXS2** | 7.02 | 296 | † |  | EF507249 |  |  |
| **PSY3** | 7.03 | 320 | † |  |  | βcry |  |
| **IPPI1** | 7.03 | 323 | † |  | AF330034.1 |  |  |
| **GGPPS2** | 7.04 | 325 | † |  | EF417574.1 |  | PC1, PC2, phytoene, phytoflu |
| **GGPPS3** | 8.01 | 326 | † |  | EF417575.1 |  |  |
| **IPPI2** | 8.03 | 348 | † |  | DN215017.1 |  |  |
| **LCYe** | 8.05 | 355 | Harjes et al. 2008 |  | NM_001153368.1 | PC1, lut, βcar, βcar, | αcar, lut |
| **PSY2** | 8.07 | 363 | † |  |  | PC3, βcar, βcry, lut |  |
| **DXS3** | 9.02 | 373 | † |  | HQ113384.1 |  |  |
| **CCD1** | 9.07 | 391 | Auldridge et al 2006 | *wc* | DQ100346 | PC2, phytoene | PC1, PC2, phytoene, phytoflu, βcry, zea, zeino, lut |
| **ZEP2** | 10.04 | 413 | † |  | CO532283.1 |  | βcry |
| **hyd3/ crtRB1** | 10.06 | 415 | Vallabhaneni et al. 2009, Yan et al. 2010 |  | NM_001112437 | PC2, phytoene, phytoflu, βcry, zea | PC3, βcar, βcry, zea |

**Supplemental Table 7**

**Supplemental Table 7: Map environment of carotenoid biosynthesis genes in *Zea mays* and clusters of carotenoid QTL identified in this study.** Pathway enzyme abbreviations are listed in Fig. 1.1 caption. Trait QTL codes are: lutein (lut), zeaxanthin (zea), zeinoxanthin (zein), β-cryptoxanthin (βcry), α-carotene (αcar), β-carotene (βcar), total colored carotenoid (total), phytoene (phyene) and phytofluene (phyflu). Trait QTL from MX analyses collocated with pathway enzymes are listed in table; trait QTL from DEexp x CI7 IL analyses that overlap with MX results are indicated by: *, IL QTL for βcry & zea; **, IL QTL for PC1. Listed gene environment corresponds to MaizeGDB database. ^†^, Genes referenced in Vallabhaneni and Wurtzel (2009).
