# Supplementary figures and images for: Genetic architecture controlling variation in grain carotenoid composition and concentrations in two maize populations
Source: Theor Appl Genet. 2013 Sep 17;126(11):2879–95. doi: 10.1007/s00122-013-2179-5 (PMC3825500; doi:10.1007/s00122-013-2179-5)

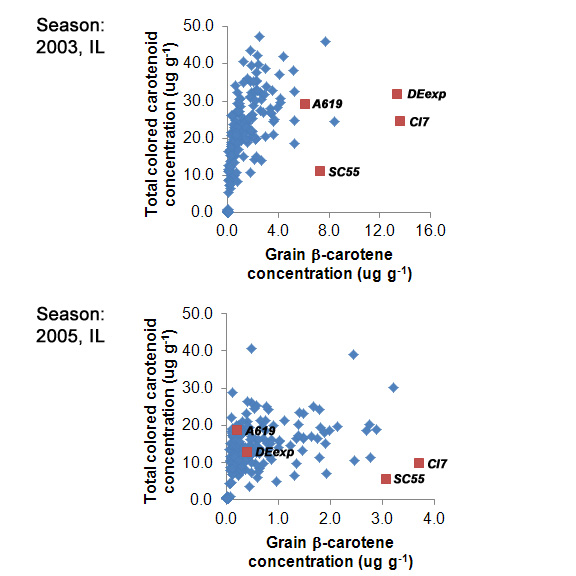

Supplement: Supplementary file 2 — Supplemental Material 2 (JPG 108 kb) [file 122_2013_2179_MOESM2_ESM.jpg]

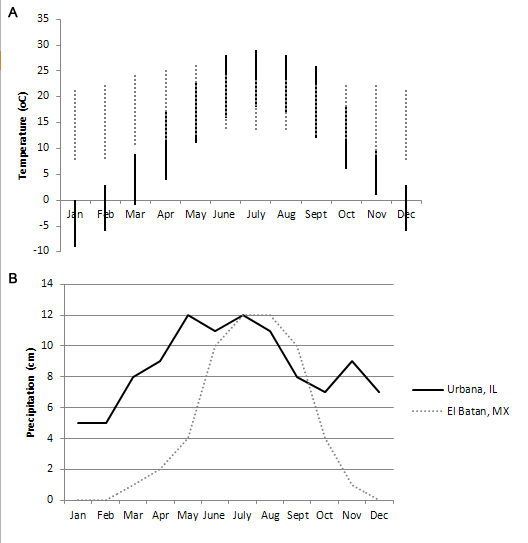

Supplement: Supplementary file 3 — Supplemental Material 3 (JPG 99 kb) [file 122_2013_2179_MOESM3_ESM.jpg]

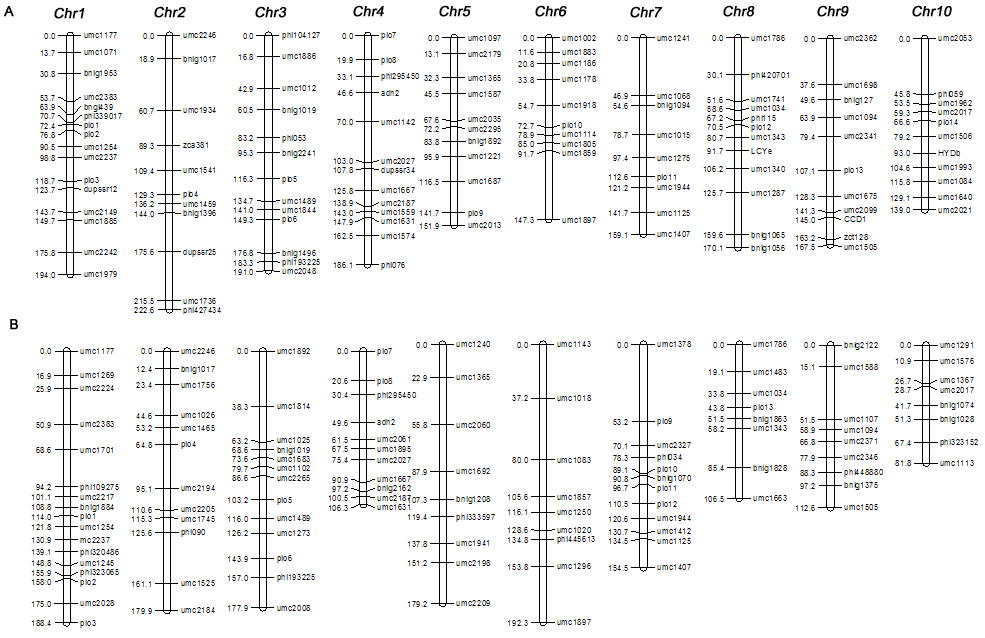

Supplement: Supplementary file 4 — Supplemental Material 4 (JPG 505 kb) [file 122_2013_2179_MOESM4_ESM.jpg]

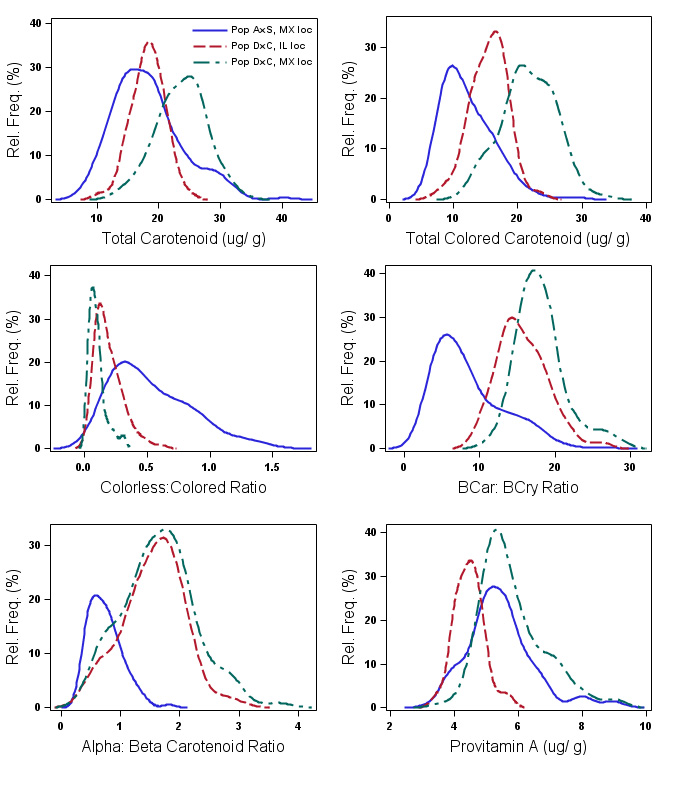

Supplement: Supplementary file 5 — Supplemental Material 5 (JPG 156 kb) [file 122_2013_2179_MOESM5_ESM.jpg]

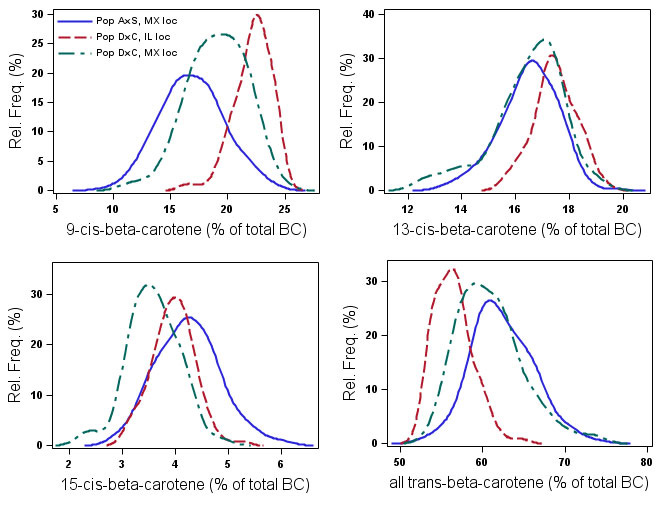

Supplement: Supplementary file 6 — Supplemental Material 6 (JPG 105 kb) [file 122_2013_2179_MOESM6_ESM.jpg]

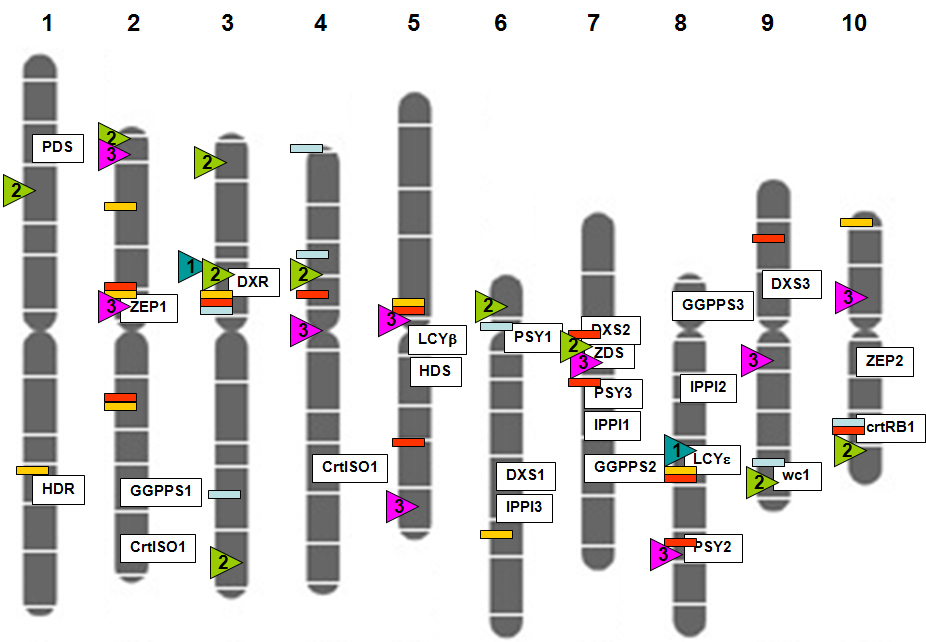

Supplement: Supplementary file 7 — Supplemental Material 7 (JPG 217 kb) [file 122_2013_2179_MOESM7_ESM.jpg]

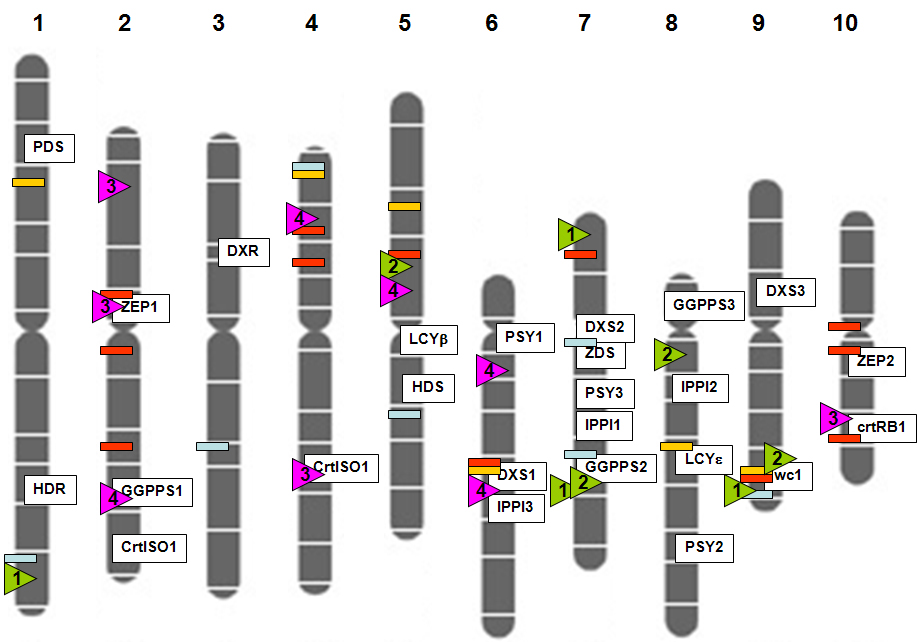

Supplement: Supplementary file 8 — Supplemental Material 8 (JPG 213 kb) [file 122_2013_2179_MOESM8_ESM.jpg]
